# Wet and dry extremes reduce arthropod biomass independently of leaf phenology in the wet tropics

**DOI:** 10.1111/gcb.16379

**Published:** 2022-09-14

**Authors:** Felicity L. Newell, Ian J. Ausprey, Scott K. Robinson

**Affiliations:** ^1^ Florida Museum of Natural History & Department of Biology University of Florida Gainesville Florida USA; ^2^ Division of Conservation Biology Institute of Ecology and Evolution, University of Bern Bern CH‐3012 Switzerland

**Keywords:** desiccation, insect biomass, leaf phenology, precipitation extremes, rainfall gradient, tropical montane cloud forest, tropical phenology, vapor pressure deficit

## Abstract

Warming temperatures are increasing rainfall extremes, yet arthropod responses to climatic fluctuations remain poorly understood. Here, we used spatiotemporal variation in tropical montane climate as a natural experiment to compare the importance of biotic versus abiotic drivers in regulating arthropod biomass. We combined intensive field data on arthropods, leaf phenology and in situ weather across a 1700–3100 m elevation and rainfall gradient, along with desiccation‐resistance experiments and multi‐decadal modelling. We found limited support for biotic drivers with weak increases in some herbivorous taxa on shrubs with new leaves, but no landscape‐scale effects of leaf phenology, which tracked light and cloud cover. Instead, rainfall explained extensive interannual variability with maximum biomass at intermediate rainfall (130 mm month^−1^) as both 3 months of high and low rainfall reduced arthropods by half. Based on 50 years of regional rainfall, our dynamic arthropod model predicted shifts in the timing of biomass maxima within cloud forests before plant communities transition to seasonally deciduous dry forests (mean annual rainfall 1000–2500 mm vs. <800 mm). Rainfall magnitude was the primary driver, but during high solar insolation, the ‘drying power of air’ (VPD_max_) reduced biomass within days contributing to drought related to the El Niño‐Southern Oscillation (ENSO). Highlighting risks from drought, experiments demonstrated community‐wide susceptibility to desiccation except for some caterpillars in which melanin‐based coloration appeared to reduce the effects of evaporative drying. Overall, we provide multiple lines of evidence that several months of heavy rain or drought reduce arthropod biomass independently of deep‐rooted plants with the potential to destabilize insectivore food webs.

## Graphical abstract

Although two‐thirds of terrestrial vertebrates consume insects and spiders, how changing rainfall regimes will affect arthropods remains poorly understood. Using spatiotemporal variation in tropical montane climate as a natural experiment, we show arthropod biomass maxima at intermediate rainfall as 3 months of both wet and dry extremes reduced arthropods by half. Integrating field estimates with local and regional rainfall to model biomass over 50 years, our intermediate rainfall model predicted locally asynchronous arthropod phenology across low‐latitude mountains. Rapid response of arthropods to increasing rainfall extremes could have cascading effects on terrestrial food webs and a range of ecosystem functions.

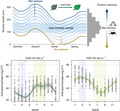

## Resumen

El aumento de las temperaturas está incrementando los extremos de precipitación, pero las respuestas de los artrópodos a las fluctuaciones climáticas siguen siendo poco conocidas. Aquí, utilizamos la variación espaciotemporal en el clima montano tropical como un experimento natural para comparar la importancia de los factores bióticos versus abióticos en la regulación de la biomasa de artrópodos. Combinamos datos de campo intensivos de artrópodos, fenología de las hojas y clima in situ a lo largo de un gradiente altitudinal de 1700 a 3100 m y un gradiente de precipitación, junto con experimentos de resistencia a la desecación y modelos multi‐decenales. Encontramos evidencia limitada para los factores bióticos con aumentos débiles en algunos taxones de herbívoros en arbustos con hojas nuevas, pero no hubo efectos a escala de paisaje en la fenología de la hoja, que rastreaba la luz y la cubierta de nubes. En cambio, las precipitaciones explicaron la amplia variabilidad interanual con una biomasa máxima en precipitaciones intermedias (130 mm mes^−1^), ya que los tres meses de precipitaciones altas y bajas redujeron los artrópodos a la mitad. Basándose en 50 años de precipitación regional, nuestro modelo dinámico de artrópodos predijo cambios en el momento de los máximos de biomasa dentro del bosque nuboso antes de que las comunidades de plantas hicieran la transición al bosque seco estacional caducifolio (precipitación media anual 1000–2500 mm vs. <800 mm). La magnitud de las lluvias fue el principal factor, pero durante la alta insolación solar, el “poder de secado del aire” (VPD_max_) redujo la biomasa en cuestión de días, lo que contribuyó a la sequía relacionada con El Niño‐Southern Oscillation (ENSO). Destacando los riesgos de la sequía, los experimentos demostraron la susceptibilidad de toda la comunidad a la desecación, excepto en el caso de algunas orugas en las que la coloración a base de melanina parece reducir los efectos de la desecación por evaporación. En resumen, proporcionamos múltiples líneas de evidencia de que varios meses de fuertes lluvias o sequías reducen la biomasa de artrópodos independientemente de las plantas de raíces profundas con el potencial de desestabilizar las redes alimentarias de los insectívoros.

## 1 INTRODUCTION

Changing rainfall patterns may affect terrestrial insects (Chown et al., 2011), as well as the food webs they support (McCluney, 2017). Although most studies of ectotherms have focused on the importance of thermal tolerances in responses to climate change (e.g. Deutsch et al., 2008; Ma et al., 2021), warming temperatures are intensifying the hydrologic cycle (Held & Soden, 2006) exposing organisms to extremes of both heavy rain and drought. For insects and spiders (arthropods) with short generation times, understanding the effects of a rapidly changing climate can begin by quantifying responses to climatic extremes on shorter time scales (Grøtan et al., 2012). However, factors driving seasonal and interannual variability in arthropod abundance at low latitudes remain unclear, including hypotheses for both biotic and abiotic regulation of population growth rates (Grimbacher & Stork, 2009; Kishimoto‐Yamada & Itioka, 2015; Wolda, 1978a, 1988, 1992).

An oft‐cited hypothesis is that numbers of herbivorous insects increase with leaf flush and the growth of new leaves, including in wet, humid systems such as tropical rainforest (Didham & Springate, 2003; Fogden, 1972; Wardhaugh, 2014; Wolda, 1978b). Bottom‐up processes predict links between producers and primary consumers as resource pulses filter up through food webs mediated by plant growth. Explanations for biotic drivers of changes in arthropod abundance relate to leaf‐chewer preferences for new leaves, which tend to be less tough (Aide & Londoño, 1989; Coley, 1983) and have higher nitrogen concentrations (Aide & Londoño, 1989; Basset, 1991; Mattson, 1980). Large arthropod taxa such as Orthoptera and Lepidoptera larvae then provide important resources for higher‐order consumers such as breeding birds (Greenberg, 1995; Newell et al., 2014). Insects that depend on plants also obtain water from their diet, for example crickets switch to moist green leaves when water is scarce (McCluney & Sabo, 2009). Thus, biotic drivers such as resource availability (Didham & Springate, 2003; Wardhaugh, 2014) might be more important than abiotic drivers such as water limitation. Multiple studies have shown either greater numbers of herbivorous insects on plants with new leaves (Basset, 1991, 1996, 2001; Wardhaugh, 2014) or peaks in abundance of herbivorous insects that coincide with community leaf flush, although also with other abiotic drivers (Boinski & Fowler, 1989; Fogden, 1972; Itioka & Yamauti, 2004; Valtonen et al., 2013). Because arthropods may be influenced by the same suite of abiotic drivers which initiate leaf flush (Wright & van Schaik, 1994), direct versus indirect pathways remain difficult to disentangle.

In the tropics, the primary abiotic hypothesis is that rainfall drives seasonality, including regulating seasonal and interannual variation in arthropod numbers (Pinheiro et al., 2002; Tauber et al., 1998; Wolda, 1978a, 1988). A phenomenon can be considered seasonal if it ‘predictably occurs at roughly the same time of year’ (Wolda, 1988), and meta‐analysis of 22 studies found regular seasonal patterns are common in tropical dry forest where maintaining water balance (intake vs. loss) becomes challenging (Kishimoto‐Yamada & Itioka, 2015). However, whether seasonal timing of rainfall maxima/minima or rainfall magnitude (cumulative amount) influence arthropods remains unclear. Additionally, arthropods can be affected by vapor pressure deficit (VPD) or ‘the drying power of the air’ (Bujan et al., 2016; Canals et al., 2015; Dias et al., 2013). Small size with high surface‐area‐to‐volume ratio increases susceptibility to desiccation from cuticular water loss (Hadley, 1984). Xeric adaptations include larger body size with ‘waxy’ cuticular lipids (Hadley, 1984), whereas in moist systems arthropods often have limited adaptations to reduce water loss (Canals et al., 2015; Dias et al., 2013). Additionally, interspecific variation contrasts with shared traits which reflect broad climatic adaptations (Hadley, 1994). For example, desiccation resistance of lowland rainforest ants differs depending on size and use of understory versus drier canopy microhabitats (Bujan et al., 2016). The role of trait variation in taxa or species‐specific responses to abiotic drivers and subsequent effects on biomass remain unclear.

In this study, we focused on arthropod biomass to provide insight into how changing rainfall regimes may affect the function of terrestrial food webs. We employed 5 years of spatiotemporal rainfall variation in the tropical Andes as a natural experiment to compare the importance of biotic versus abiotic drivers with limited effects of photoperiod. To disentangle bottom‐up effects across food webs, we focused on the resource availability hypothesis and tested the following predictions: (H1.1) plants with new leaves support greater numbers of arthropods, (H1.2) primarily herbivorous taxa increase with leaf flush, especially large leaf‐chewers such as Orthoptera and Lepidoptera larvae, and (H1.3) plant‐arthropod peaks synchronize across local landscapes. For abiotic drivers, we predicted limited elevational effects because of weak temperature seasonality (<2°C; Newell et al., 2022c), and thus we focused on precipitation. To isolate the direct effects of rainfall from bottom‐up processes mediated by plant growth, we expected independent responses of arthropods and plants to abiotic drivers, and we tested the following predictions of the water limitation hypothesis: (H2.1) arthropod maxima/minima are seasonal with regular dry season reductions in biomass across a rainfall gradient, (H2.2) rainfall magnitude drives spatiotemporal fluctuations in biomass, and/or (H2.3) high diurnal VPD reduces arthropod biomass. We expected VPD to be important because of poor physiological tolerances for dry conditions in tropical moist forest, and we used desiccation resistance experiments to test the following prediction: (H2.4) reduced humidity impacts survival time of cloud forest arthropods across taxa supporting community‐wide effects of evaporative drying on biomass. To understand consequences for insectivores (e.g., birds that primarily consume insects and other invertebrates), we used results of the previous analysis to build a dynamic model to characterize long‐term phenological means for arthropod biomass in humid montane cloud forest landscapes. We also discuss implications for cascading effects of changing rainfall on insectivore populations.

## 2 METHODS

### 2.1 Tropical montane cloud forest network

**FIGURE 1:**
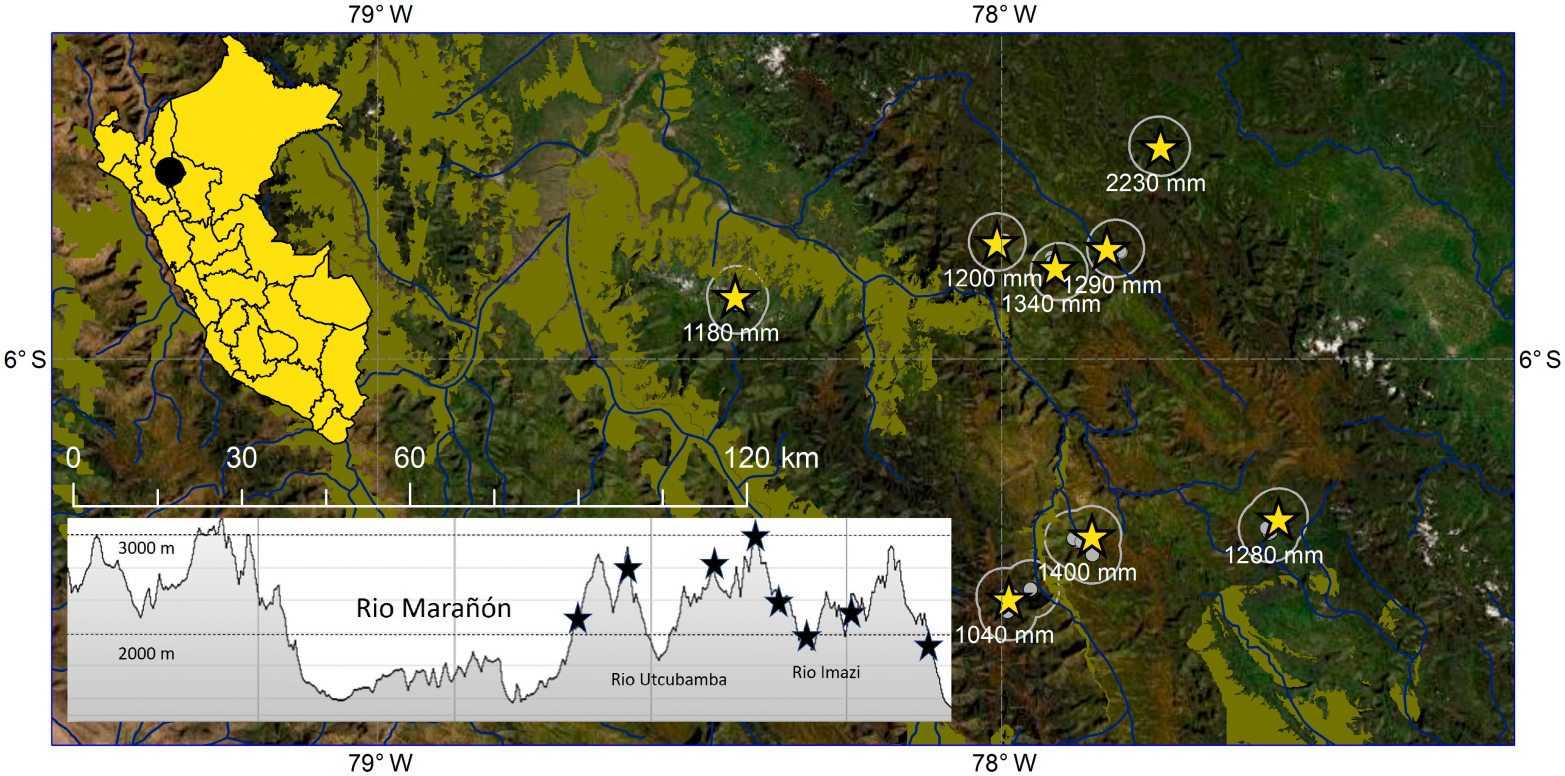
Complex elevational gradient at 5–7° S in the Andes of northern Peru used as a natural experiment to model arthropod biomass across a rainfall gradient. Weather data were collected for each landscape from 2015 to 2019 using in situ rain gauges (yellow stars), arthropod and leaf phenology data were collected at 3–9 subsites per landscapes (gray points), and we used vegetation greenness from the MODIS Terra satellite within a 5‐km radius (white line) filtered for >50% forest cover within a 300‐m elevation band. Horizontal cross section shows landscapes by elevation across four watersheds. Landscapes labeled by mean annual rainfall estimated over 50 years. Dry forest biome shown in green‐brown (Josse et al., 2009).

We studied spatiotemporal variation in arthropod biomass across a gradient of elevation and rainfall between 5–6° S and 77–78° W in the Andes of northern Perú. A network of eight cloud forest landscapes were located 10–100 km apart across a 10,000‐km^2^ area (Figure 1). Concurrent in situ weather data were collected for each landscape beginning in 2015–2016. Spanning a 1700–3100 m elevational gradient, temperatures inside forest averaged 10–17°C (8–15°C *T*_min_, 13–20°C *T*_max_). Rainfall decreased from the eastern slopes to inter‐Andean ridges with mean annual rainfall 1000–2500 mm year^−1^ (interannual range 800–3000 mm). Cloud forests were characterized by high cloud cover (annual means 65%–81%) and humidity (daytime means ≥90% inside forest). Across a complex elevational gradient spanning four watersheds moisture regimes did not covary with elevation (Newell et al., 2022c).

**FIGURE 2:**
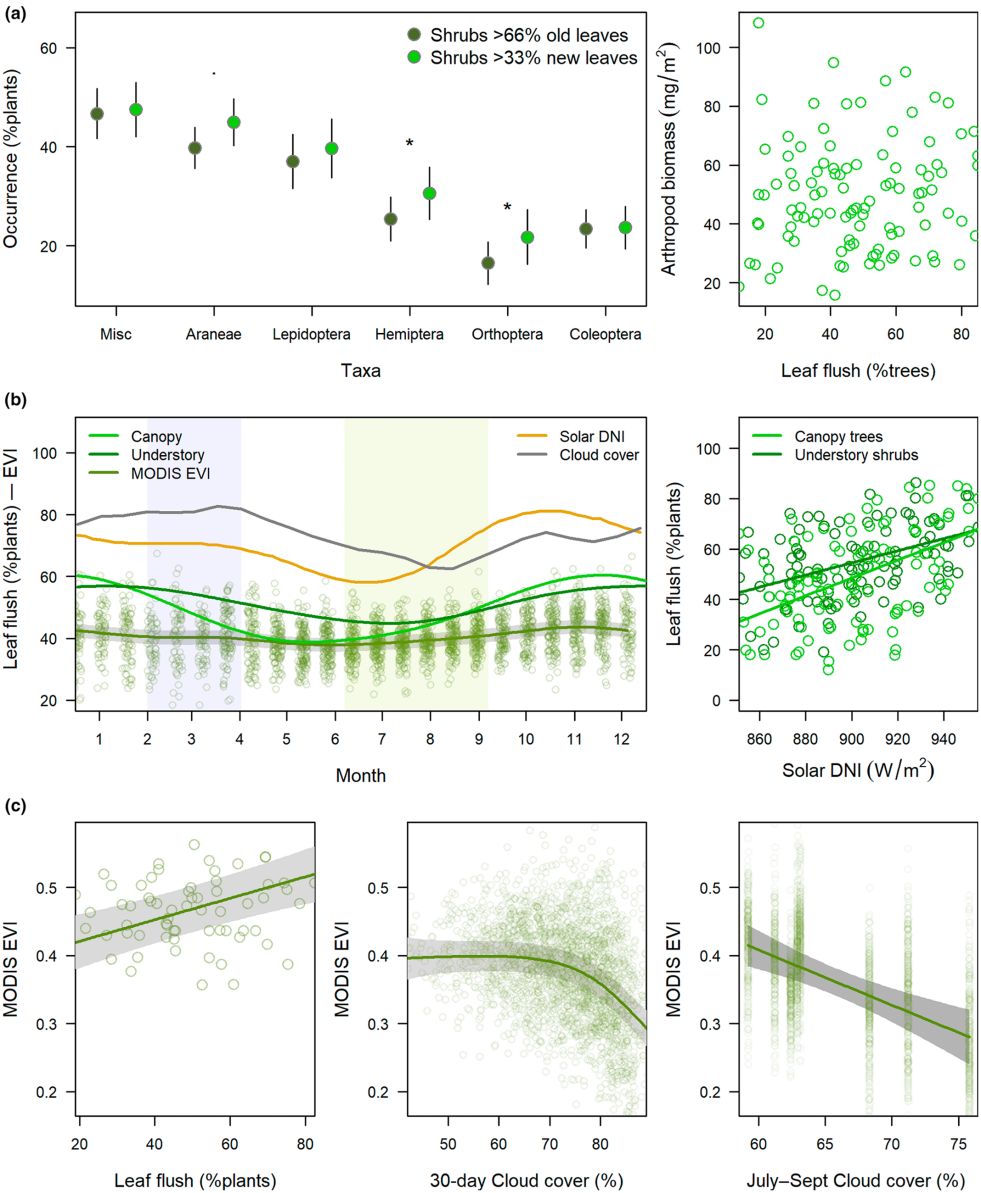
Relationship of foliage arthropods to leaf phenology across scales in evergreen cloud forest at 5–7° S in the Andes of northern Peru. (a) Occurrence of arthropod taxa on individual shrubs with new/initiating versus old leaves and landscape‐scale arthropod biomass by canopy leaf phenology (116 visits, 8 landscapes, 2015–2019). Error bars represent *SE* with asterisks for standard *p*‐values; Lepidoptera represent larvae. (b) Seasonal leaf phenology relative to abiotic drivers. Direct normal irradiance (DNI) was the top model for field observations with the strongest effect on canopy trees. (c) Weakly related to field observations, the top model for the enhanced vegetation index (EVI) included monthly plus dry season (July–September) cloud cover; partial residual plots control for decreasing EVI with elevation (Figure S2).

Typical of a humid highland climate (Köppen: *Cfb*), rain occurred year‐round with 9%–15% falling during the dry season (July–September) and 18%–36% in other quarters of the year. July was the driest month (interannual range 20–100 mm) and March the wettest month (interannual range 120–500 mm) with moderate rainfall including both wet and dry extremes (Newell et al., 2022c). Rainfall seasonality varied by less than 3 weeks among sites (Newell et al., 2022c), and at this latitude daylength changed by 41–46 min. Maximum VPD (VPD_max_) increased with solar insolation after the winter solstice (Figure 2a; Figure S1) and the driest air occurred during the austral winter/spring when temperatures warmed although monthly means for VPD_max_ remained <0.7 kPa. See Supporting Information for additional details on processing weather data and Newell et al. (2022c) for further information on cloud forest climate in the region including a drought in 2016 related to the El Niño‐Southern Oscillation (ENSO).

### 2.2 Spatiotemporal 5‐year field study

In each landscape, we sampled arthropods and leaf phenology at 3–9 sub‐sites <5‐km apart within a 300‐m elevation band. Sub‐sites were divided among contiguous (*n* = 27) and fragmented forest (*n* = 20) as part of a broader study examining the interaction of climate and land‐use on biodiversity (Ausprey et al., 2021; Newell, 2021). Fragments which included arthropod sampling primarily ranged from 3 to 30 ha in size, with two visits to 90‐ha fragments. Sampling at contiguous forest focused on accessible areas. Matrices surrounding fragments were characterized by mixed agriculture and pasture (Ausprey et al., 2021). Most sub‐sites within each landscape included repeated sampling at different times of year (2–10 visits) with a single visit to the least accessible sites. Cloud forest plant communities varied by elevation with common trees including *Alnus*, *Axinaea*, *Brachyotum*, *Cecropia*, *Cedrela*, *Ficus*, *Heliocarpus*, Lauraceae spp., *Schefflera*, *Siparuna*, *Solanum*, *Urera*, *Vallea*, and *Weinmannia*; and common understory shrubs including *Chusquea*, *Hedyosmum*, *Miconia*, *Palicourea*, *Piper* and *Psychotria*,

To examine variability across space and time, fieldwork was conducted at different times of year during six sampling ‘snapshots’ (3–6 months each) from 2015 to 2019 (Figure S1). During each sampling ‘snapshot’, landscapes were visited consecutively every 1–2 months for year‐round coverage in all months except March–April when landslides and road closures made travel unsafe; fieldwork was also reduced on the eastern slopes due to safety concerns. Field data were collected during the dry and transition dry‐to‐wet seasons for 3 years in June/July–November 2015–2017. Additional transition wet‐to‐dry season data were collected for 2 years in May–July 2018–2019, and we collected data for one wet season in December–February 2018/19.

#### 2.2.1 Leaf phenology

We collected ground‐based data on canopy and understory leaf phenology combined with vegetation greenness from the MODIS Terra satellite. On each landscape‐visit, we scored in situ leaf flush for ≥100 individuals of both canopy trees and understory shrubs beginning in 2016. Shrubs were scored at five points located 30‐m apart along transects, and we recorded phenology for the 20 nearest shrubs (0.5–15 m tall). Trees were scored in the field using binoculars at canopy vantage points, as well as documented using repeat photographs (Figure S2). We recorded plant phenophases as leafless, initiating, new leaves or old leaves. Categorical scoring was used for rapid field data collection, and we defined categories covering >33% of the plant to focus on periods of extensive leaf growth including continuously leafing species. We classified developing leaves that were still unfurling as initiating, while new leaves were distinguished from old leaves based on bright green coloration as well as leaf wear (Figure S2). Field leaf phenology was scored by F.L.N.

For satellite data, we used the enhanced vegetation index (EVI) for high biomass regions to examine vegetation greenness within a 5‐km radius of our study sites from 2001 to 2019. We used 16‐day MODIS EVI at 250 m resolution (MOD13Q1v6) and selected pixels in a 300‐m elevation band with >50% forest cover (Hansen et al., 2013). Data were filtered to remove cloud cover (Samanta et al., 2010) and we used 6‐weeks running medians weighted by usefulness scores. For the full dataset comparing biotic and abiotic models, EVI data were used to complete ground‐based phenology during study initiation in 2015, and for missing data due to cloud cover we used monthly normals by landscape averaged across available years. See Supporting Information for additional details on processing satellite data.

#### 2.2.2 Arthropod transects

We used modified branch beating to sample a range of foliage taxa (Ozanne, 2005), as well as aerial sweep netting and leaf‐litter quadrants to compare different forest strata (Cooper & Whitmore, 1990). Branch beating was selected to rapidly capture a range of foliage arthropods using a single method. Less time consuming than visual counts, foliage branch beating detects 50%–70% of caterpillars (Bodner, 2011), and we also captured active taxa by beating over a sweep net which could be rapidly closed. Arthropods were systematically sampled at 30‐m intervals along semi‐randomly placed transects. Each visit to a landscape included 20 branch‐beatings (~2 h), 20 sweep‐nets (~1 h), and 10 leaf‐litter quadrants (~1 h); half were collected by the same observer (F.L.N.) and the remainder by 1–2 trained technicians who varied by sampling period. We counted arthropods by order recording size to the nearest mm (length × width). For each plant we recorded leaf phenophases as new/initiating or old leaves (Figure S2). We calculated arthropod biomass (dry mass) using published taxa‐specific regression coefficients primarily from the humid tropics (Gruner, 2003; Wardhaugh, 2013; Table S1). Results are presented as mg/m^2^ (two samples) to facilitate comparison across studies. See Supporting Information for additional details on arthropod sampling methods.

#### 2.2.3 Statistical analysis of field data

We used an Akaike information criterion (AIC) model selection approach to examine support for different biotic and abiotic models to explain changes in arthropod biomass (Burnham & Anderson, 2002). We conducted two analyses at different spatial scales by sample (individual plant) and landscape‐visit in which we summed biomass or counts >3 mm per visit with number of samples (10–20) as an offset. Data were analyzed using generalized linear or additive mixed models with the gamm4 package (Wood & Scheipl, 2020) in program R which integrates polynomial smoothing functions from mgcv (Wood, 2017) with random effects for a variety of distributions using maximum likelihood methods from lme4 (Bates et al., 2015). Arthropod biomass was modeled using a Gamma distribution with a log link function and results were back transformed for interpretation of effect size. To control for spatiotemporal autocorrelation, we used crossed random intercepts with repeated measures for space and time. Random effects by sample included observer, sampling period, and plant group crossed with a nested sampling design (1| landscape/visit). Random effects by visit were simplified to sampling period and landscape; the same random structure was used to analyze landscape‐scale leaf phenology by landscape‐visit. Models were visualized using partial regression plots with the visreg package (Breheny & Burchett, 2017) and we calculated means using the emmeans package (Lenth, 2020) with asymptotic or bootstrapped 95% confidence intervals (CI). For linear relationships, we also examined structural equation modeling (SEM) fitted with a gaussian distribution using the R package piecewiseSEM (Lefcheck, 2016).

We examined the influence of leaf phenology on arthropods at different scales, as well as abiotic drivers of regional leaf phenology. (1) For the individual plant (sample), we examined both total biomass and the occurrence of common arthropod taxa on shrubs with new versus old leaves. For occurrence we used a binomial logistic regression (yes/no); results were similar for arthropod counts with a poisson distribution, but convergence was an issue for several taxa, so we present occurrence results. (2) We examined the relationship between arthropod biomass and leaf phenology (ground observations and EVI) at the scale of the landscape‐visit. To model abiotic drivers of leaf flush, we used a negative binomial distribution for over‐dispersed count data with theta estimated from a model without random effects; results were similar with a continuous Gamma distribution. We used the number of plants with >33% new/initiating leaves as the response variable and the number of plants observed as an offset. For EVI we used a gaussian distribution which provided the best fit to the data.

We examined short‐term effects of weather on arthropod activity at the scale of the sample and focused on abiotic drivers primarily at the scale of the landscape‐visit which greatly reduced the variance. (1) At the level of the sample, we examined time of day, temperature, and wet vegetation as samples were typically collected at different times (Figure S4) over several days around other field work during a landscape‐visit. We also examined fragmentation and elevation by sample as, depending on field logistics, we sometimes sampled arthropods at several sub‐sites within a landscape on the same visit. (2) We examined responses to longer‐term climatic conditions at the scale of the landscape‐visit. First, we used AIC model selection to compare different rainfall accumulation and mean VPD_max_ time lags (5–90 days). Top models were included in the final model set comparing different weather variables with leaf phenology. We also examined interactive models, but to simplify interpretation, we only present interactions ranked better than an additive model. We also ran analyses excluding aposematic taxa, but results were similar, and we present the full dataset.

### 2.3 Desiccation resistance experiments

#### 2.3.1 Experimental design

We used a simple experiment to measure susceptibility to desiccation across cloud forest arthropod communities. For five common foliage taxa (Araneae, Orthoptera, Phasmida, Coleoptera, Lepidoptera larvae), we examined effects of reduced humidity on survival time without access to food or water (Bujan et al., 2016). We conducted experiments on a random sample of >400 individuals >3 mm in length which we collected with foliage branch beating on 48 different landscape visits, 2017–2019 (Table S8). Individual arthropods were isolated from contact in 2–5 ml plastic vials labeled with a unique identifier and covered with cheese cloth. We identified individuals to order, recorded color and measured each arthropod (length × width) to calculate surface‐area‐to‐volume ratio assuming a cylindrical shape.

For experiments, individual arthropods were randomly assigned to one of three sealed plastic containers (>10,000 ml with adequate oxygen) with different levels of humidity (Figure S10). A range of humidity was manipulated by placing 100 ml of silica inside each chamber to represent dry (new silica), intermediate (partially used silica) or wet conditions (fully used silica); the high humidity treatment included 100 ml of water and served as a control (Figure S10a). Temperature and relative humidity (RH) were recorded every 15 min by Hobowear data loggers (U23‐001) located inside each chamber. Chambers were maintained at ambient temperatures typical of daytime highs in cloud forest (~18°C) with mean RH of 40%, 74% and 92%, respectively (Figure S10b,c).

#### 2.3.2 Statistical analysis of experiments

Models were fit using a Gamma distribution with a log‐link function which provided the best fit to the data. For experiments we included individual surface‐area‐to‐volume ratio as a covariate to control for body size. We calculated VPD from temperature and RH using the package *psychrolib* (Meyer & Thevenard, 2019). For each arthropod we calculated mean VPD for days the individual was alive as the predictor, and survival time as the response variable. We also examined RH, but including temperature provided a better fit to the data and we present results for VPD. Results are presented for *p*‐values <.05. See Supporting Information for additional details on design and analysis of desiccation resistance experiments.

### 2.4 Dynamic multi‐decadal model

We integrated results from our analysis of arthropod field data with local and regional rainfall to dynamically model monthly arthropod biomass for each landscape over 50 years. This model combined parameter estimates from empirical data collected in the field with daily rainfall scaled to cloud forest landscapes using the normal ratio method (Newell et al., 2022c); long‐term data were obtained from regional weather stations maintained and compiled by the Peruvian National Meteorology & Hydrology Service (SENAHMI). We used a decision tree approach based on timing and extent of cumulative daily rainfall for each landscape to incorporate different time lags, linear and curvilinear relationships (Figure S11). We used monthly arthropod biomass estimated from the model to describe long‐term phenological means by landscape on multi‐decadal time scales across a montane rainfall gradient. We also examined temporal variability based on the coefficient of variation (CV). To model long‐term phenological means, we used generalized additive mixed models (GAMMs) with a cyclic cubic regression spline, initial knots set at 12 months, a temporal smooth by date, plus a random intercept by year. See Supporting Information for additional details on the arthropod biomass model.

## 3 RESULTS

We sampled arthropods on >100 visits to capture spatiotemporal weather variation across the region, including an ENSO related drought in 2016 (Figure S1). During 2424 branch beatings we counted 22,189 arthropods on foliage; during 1805 sweep nets we counted 17,255 aerial insects, and during 1007 leaf‐litter quadrants we counted 8653 ground‐dwelling invertebrates. Counts were dominated by small spiders whereas arthropods >3 mm were the primary contributor to biomass (Table S2). For all sampling methods arthropod biomass increased with both abundance (counts >3 mm), as well as arthropod length (Figure S3). Most taxa were represented by numerous individuals with a range of sizes, although Orthoptera and adult Lepidoptera contributed a greater percentage of total biomass compared to abundance (Table S2). Contributions of different orders varied among our eight landscapes, but we found no effect of elevation or forest fragmentation on total biomass for any sampling method (Table S3).

### 3.1 Indirect biotic effects mediated by plant growth

#### 3.1.1 Individual scale

When we compared shrubs with new/initiating versus old leaves there was a weak effect of leaf flush at the scale of the sample collected from an individual plant (Table S3). Consistent with the biotic hypothesis, arthropod biomass was 13% (CI 7–19) greater on shrubs with >33% new/initiating compared to old leaves. Orders that include many herbivorous taxa (Orthoptera, Phasmida, Hemiptera, Lepidoptera), contributed a third of foliage biomass at 14 mg/m^2^ (CI 9–17) compared with taxa with diverse diets at 27 mg/m^2^ (CI 22–36), and effect size increased to 23% (CI 16–30) for herbivorous taxa whereas arthropod biomass was not significantly different on new leaves when pooling diverse taxa (*t* = 0.63, *df* = 2406, *p* = .52). However, although Orthoptera and Hemiptera >3 mm in length were 20%–32% more likely to be found on shrubs with new compared with old leaves, differences were not significant for Lepidoptera larvae (Figure 2a). Branch‐beating samples from individual plants were extremely variable and none of the models by sample explained >2% of the variation.

#### 3.1.2 Landscape scale

Inconsistent with strong biotic control, none of the leaf phenology metrics (ground‐based or remote sensing) explained variation in biomass of foliage arthropods per landscape‐visit, including field counts of shrubs and canopy trees with new/initiating leaves, or vegetation greenness from the MODIS Terra satellite (Table S3; Figure 2a). Regionwide the proportion of plants with new leaves was weakly seasonal with a 14%–26% change in magnitude from maxima >60% during the austral spring (October–December) to minima of 40%–47% at the end of the rainy season (Table S4; Figure 2b). Ground‐based leaf phenology was best explained by regional solar cycles, and the top model was direct normal irradiance while trees tended to be more seasonal than shrubs (Table S4; Figure 2b). Only weakly related to field observations, top models for EVI related to cloud cover (Table S4; Figure 2c). EVI was reduced during months with >70% cloud cover as well as at sites with >70% dry season cloud cover (Figure 2c). EVI also decreased at higher elevation (Figure S2) across a complex gradient where precipitation did not covary with elevation (Newell et al., 2022c).

### 3.2 Direct abiotic effects of moisture regimes

#### 3.2.1 Rainfall accumulation

**FIGURE 3:**
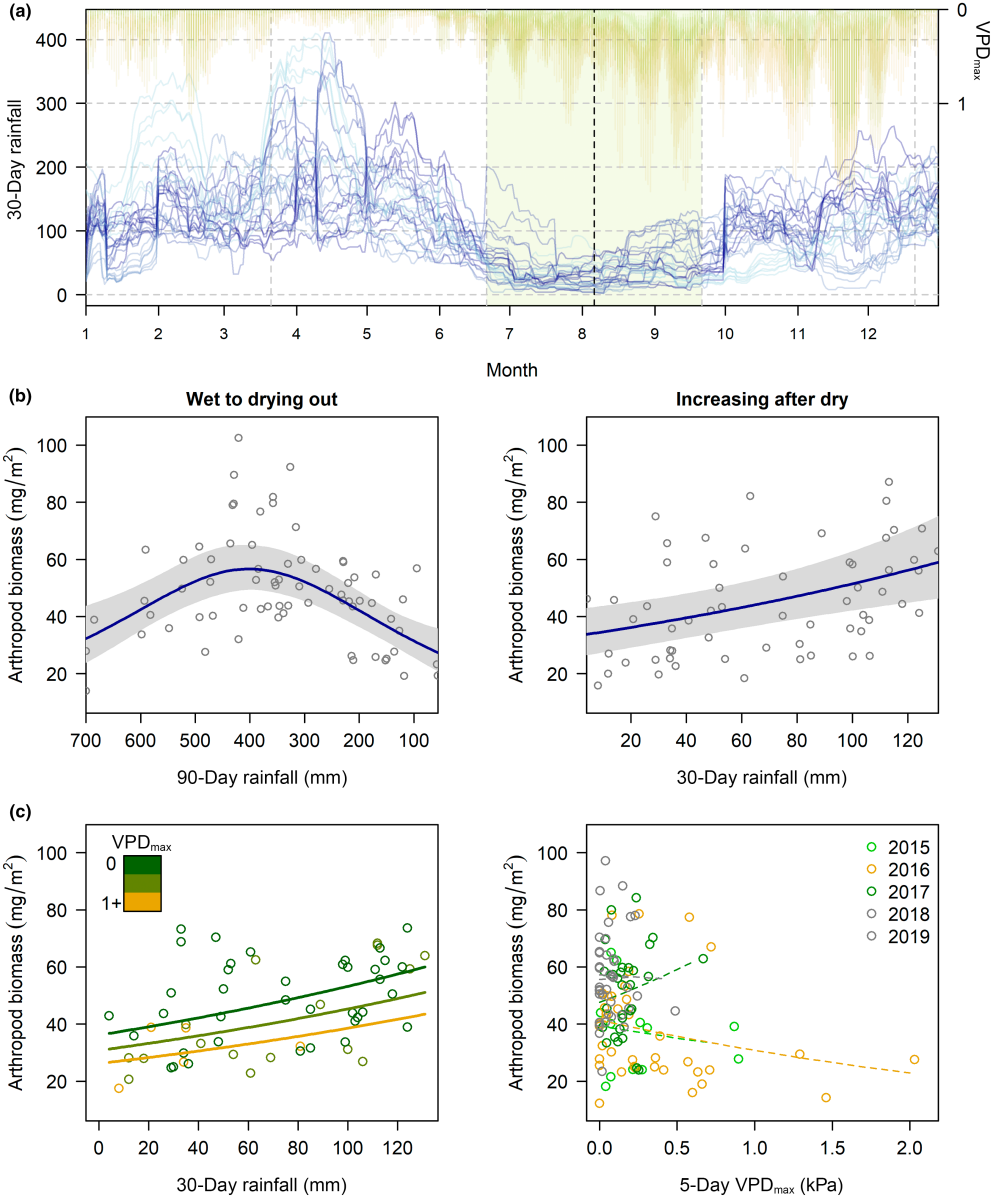
Direct effect of abiotic conditions drove a twofold change in arthropod biomass at 5–7°S in the Andes of northern Peru. (a) Spatiotemporal variation in the magnitude of seasonal rainfall (blue) and vapor pressure deficit (yellow) across eight landscapes and 5 years, 2015–2019; shaded areas indicate austral winter (dry season) with dashed line for mean lowest 30‐day rainfall regionwide. (b) Partial residual plots for rainfall accumulation time lags explaining biomass of foliage arthropods pre (70 visits) and post the dry season (58 visits).

For foliage arthropods, we found no evidence of short‐term changes in activity, and biomass was relatively stable throughout the day (Table S3; Figure S4). There was strong support for abiotic control of arthropods, but relationships with rainfall were dynamic and nonlinear. Unlike seasonal leaf phenology, Julian date did not provide a good fit to biomass of foliage arthropods (Table S3). Instead, top models related to rainfall magnitude, and including different time lags improved model fit. When we excluded increasing rain after the dry season based on visual inspection of graphs, a curvilinear response to 90‐day rainfall accumulation went from explaining 20% to 30% of the variation (Tables S3 and S5). Arthropod biomass decreased after extended heavy rain and as cloud forest dried out with biomass maxima at intermediate rainfall around 130 mm month^−1^ for 3 months (Figure 3b). After reduced dry season rainfall, foliage arthropods rebounded rapidly within 1 month of returning rain (Figure 3b). The top model included a linear increase with 30‐day rainfall accumulation plus negative effects of mean 5‐day VPD_max_ while controlling for weak increases by season date during the austral spring (Table S5; Figure 3b). Overall, the additive model explained 30% of the variation with 18% explained by rainfall, 8% by VPD_max_ and 7% by season date. Leaf flush provided an equivalent model to season data and may have contributed to increasing arthropod biomass after the dry season, although not at other times of year (Table S5). Structural equation modeling supported direct effects of rainfall and did not show a link between biomass and leaf phenology after the dry season (Figure S5); we were not able to examine the non‐linear relationships at other times of year due to limitations with the SEM approach (Lefcheck, 2016).

Effects of rainfall were strongest for foliage arthropods, whereas for aerial and leaf‐litter strata showing short‐term changes in activity, relationships with rainfall were weaker. Across models, rainfall predicted a twofold change in biomass of foliage arthropods from lows of 31 mg/m^2^ to highs of 61 mg/m^2^. Changes in foliage biomass were explained by a combination of both greater abundance (counts >3 mm), as well as larger individuals with similar, although weaker pattern for both metrics (Figure S6). Unlike foliage arthropods, we found short‐term changes in activity for other strata (Figure S4) and biomass was higher on dry days (aerial insects) or warm afternoons (leaf litter invertebrates) (Tables S6 and S7; Figure S7). When controlling for activity, effects of rainfall were weaker, but other strata showed similar responses to 90‐day rainfall accumulation and were also not seasonal (Tables S6 and S7; Figure S7). Most arthropod taxa showed consistent responses to rainfall (Figures S8 and S9), and both curvilinear relationships and different time lags were apparent for three common soft‐bodied foliage taxa (Araneae, Orthopteroids, and Lepidoptera larvae). Hemiptera increased with 30‐day rainfall accumulation after the dry season, Hymenoptera were reduced at lower 90‐day rainfall accumulation, but rainfall did not explain Coleoptera for any strata. See Supporting Information for detailed results from aerial sweep‐nets and leaf‐litter quadrants.

#### 3.2.2 Vapor pressure deficit

As solar insolation increased in the austral spring, VPD contributed a significant additive effect to rainfall (Table S5), although VPD alone explained only 4% of the variation (Table S3). During periods of low to increasing rainfall (<130 mm month^−1^), a 1 kPa increase in mean 5‐day VPD_max_ predicted a rapid 27% reduction in biomass of foliage arthropods (Figure 3c). Because cloud forest rarely dried out, exact time lags were difficult to identify, but 5‐day VPD_max_ provided a better fit to the data than either daily or 10‐day means. Dry, sunny weather explained reductions in foliage arthropods during an ENSO‐related drought in 2016 when biomass, although locally variable, was 21% lower during the austral spring than at the same time in other years (Figure 2c). We did not find a relationship with VPD for arthropods in other strata (Tables S6 and S7) either because of reduced sampling, confounding effects of activity on dry days (aerial insects) or slower heterogenous rates of drying (leaf litter).

### 3.3 Desiccation resistance experiments

**FIGURE 4:**
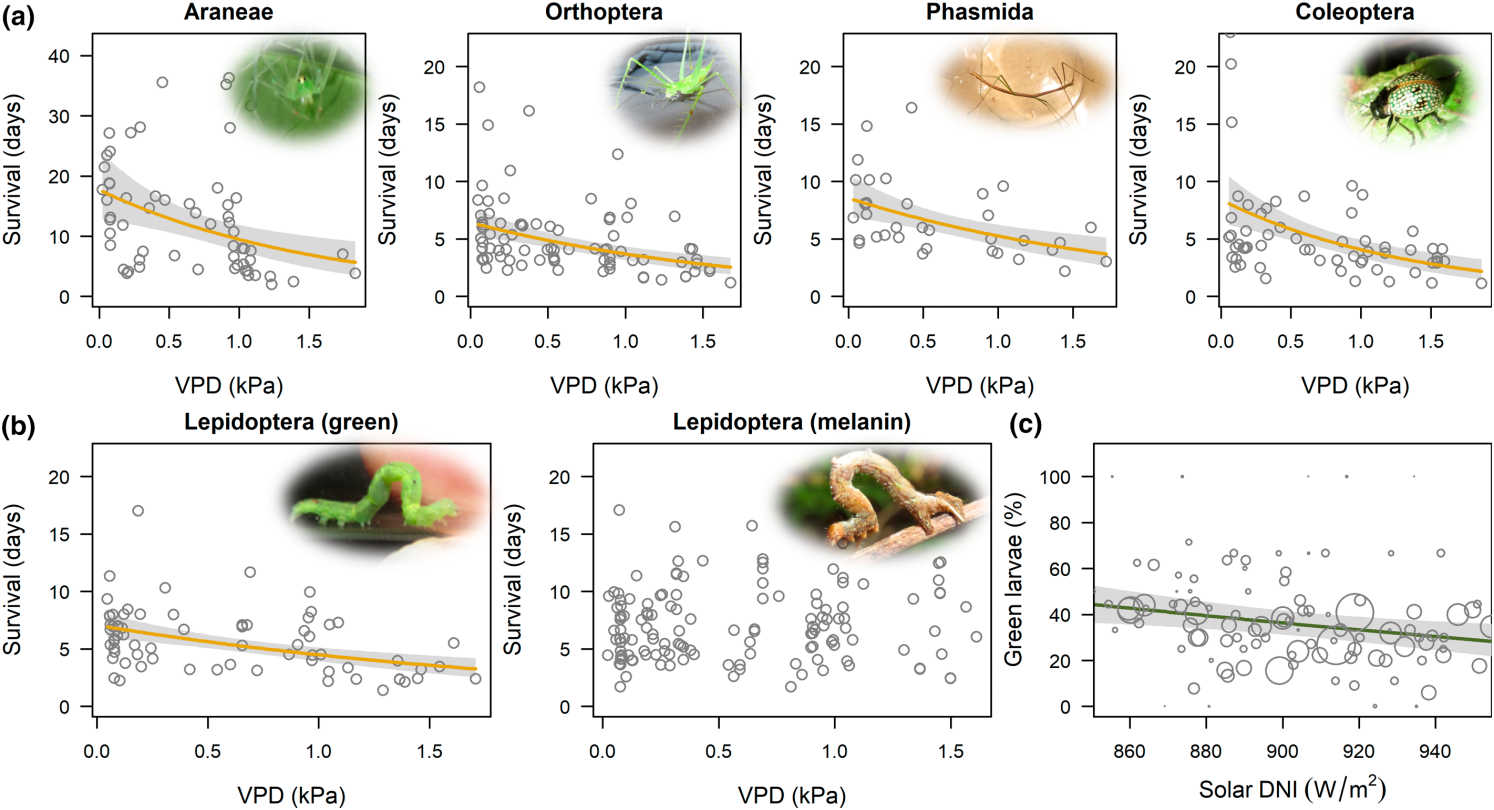
Desiccation resistance experiments across humid‐adapted cloud forest arthropods (*n* = 477 individuals). (a) Common foliage taxa were negatively affected by high vapor pressure deficit (VPD) or the ‘drying power of the air’. (b) except for Lepidoptera larvae with reduced effects of VPD on taxa with melanin‐based pigments (tan, brown or black). (c) Field data showed percentages of green Lepidoptera larvae were reduced during high solar insolation in the austral spring when VPD increased; circles scaled per 10 individuals. Regression lines from partial residual plots by order control for surface‐area‐to‐volume ratio based on size; lines shown for significant regressions (*p* < .05). Range of VPD reflect typical cloud forest maxima. Photo credits F. L. Newell.

To demonstrate a physiological basis and support direct, community‐wide effects of evaporative drying on arthropod biomass, we conducted experiments for 477 individuals from five common foliage orders (Araneae, Orthoptera, Phasmida, Coleoptera, Lepidoptera larvae). We found strong evidence that cloud forest arthropod communities were susceptible to desiccation, and reduced humidity had a significant negative effect on all foliage taxa except for Lepidoptera larvae (Table S10; Figure 4a). A 1.0 kPa increase in VPD reduced survival time 37%–52% by order, although there was substantial variation within broad taxonomic groups, and VPD explained 14%–30% of the variation. Across taxa a 34% reduction in survival time was highly significant (*t* = 8.1, *df* = 475, *p* < .001) with VPD explaining 12% of the variation. High surface‐area‐to‐volume ratio also reduced survival time 18%–30% with no size effect for Coleoptera.

Melanistic coloration appeared to play a role in desiccation resistance of cloud forest Lepidoptera larvae which were dominated by geometrids. When we examined Lepidoptera larvae by color, high VPD had a significant negative effect on caterpillars with primarily green coloration (*t* = −4.36, *df* = 66, *p* < .001), but no effect on pooled caterpillars with tan, brown or black coloration (*t* = 0.74, *df* = 136, *p* = .45; Table S10; Figure 4b). Relating experiments to field data, we captured 34% (CI 28–44) fewer green Lepidoptera larvae during high solar insolation in the austral spring with some support for a seasonal cycle and greater numbers of green caterpillars after higher 90‐day rainfall accumulation (Table S8; Figure 4c). For comparison, we examined other leaf‐chewers in which >30% of individuals on understory shrubs were primarily green. Orthoptera and Phasmida were negatively affected by high VPD for both color groups (all *p* < .05), and in foliage field samples we found no relationship between green coloration and abiotic variables, although smaller, green Phasmida were more likely to be captured on shrubs with new leaves (Table S8).

### 3.4 Dynamic multi‐decadal model

**FIGURE 5:**
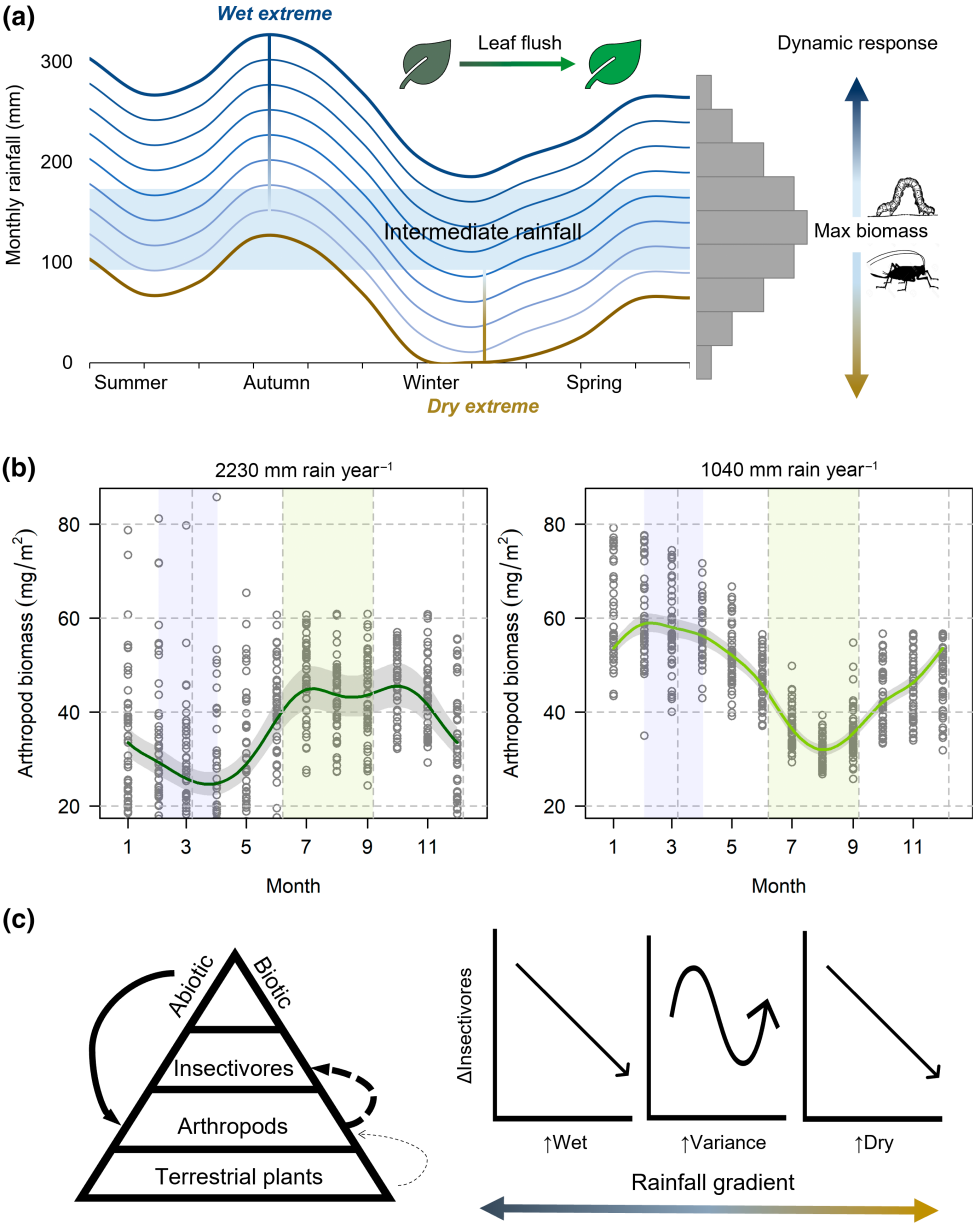
Arthropod biomass model in evergreen cloud forest at 5–7°S in the Andes of northern Peru. (a) Conceptual diagram of dynamic response of arthropods to changes in rainfall magnitude independent of leaf flush. Rainfall isotherms indicate spatiotemporal variation relative to seasonality in northern Peru and arrows indicate lagged decreases in arthropod biomass after wet and dry extremes. (b) The quantitative model predicted shifts in timing of resource maxima across a rainfall gradient based on 50 years of regional weather data; graphs for study extremes (see Figure S12 for all landscapes). (c) Rapid response of arthropods to rainfall extremes are hypothesized to destabilize insectivore populations before effects on deep‐rooted plants which access soil moisture. Both increasing rainfall in wet systems and decreasing rainfall in dry systems could reduce tropical insectivore populations while greater seasonal and interannual extremes contribute to variable population dynamics

Using regional rainfall data scaled to in situ gauges, we modeled phenological means for arthropod biomass over 50 years across our montane rainfall gradient. Dynamic responses to intermediate rainfall optima (Figure 5a) predicted dry season resource maxima at the wettest site shifting within <100 km to wet season resource maxima at the driest site (Figure 5b). Across inter‐Andean sites (1000–1400 mm year^−1^), the intermediate rainfall model predicted a range of bimodal resource peaks between wet and dry season maxima (Figure S12). At transitional sites, biomass was reduced during the dry season in some years but not others and interannual variability complicated phenology on shorter timescales. The model predicted the greatest CV for rainy season biomass at the wettest site (CV 30%), whereas dry season variation was similar across the gradient (CV 10%–20%; Figure S13). Interestingly at all but the driest site, the model indicated the most stable arthropod resource availability in June around the winter solstice (CV <10%; Figure S13). When we incorporated rainfall uncertainty on the eastern slopes (2200–3200 mm year^−1^), wet‐to‐dry season shifts in arthropod biomass maxima were accentuated by greater rainfall (Figure S14).

## 4 DISCUSSION

Using spatiotemporal variation in tropical montane climate as a natural experiment, we found that even in the wet tropics abiotic drivers regulate arthropod biomass more strongly than biotic drivers. Leaf phenology had limited effect at landscape scales, and arthropod biomass was explained by a curvilinear response to rainfall magnitude with intermediate maxima. Fluctuations around rainfall optima drove interannual variability as biomass was reduced after 3 months of both high and low rainfall. Despite similar seasonality, our dynamic multidecadal model showed how differences in local rainfall accumulation can result in heterogenous arthropod phenology at small spatial scales across low‐latitude mountains. Combined observational and experimental evidence highlight negative effects of evaporative drying (VPD_max_) on humid‐adapted arthropod communities, although we also found evidence for trait variation related to melanin. Bottom‐up processes are an important contributor to endotherm responses to rainfall (Boyle et al., 2020), and our results suggest increasing rainfall extremes could destabilize insectivore food webs before effects on long‐lived plant communities which access soil moisture through deep roots.

This is the first study to quantitively examine the importance of leaf phenology for foliage arthropods at different scales across a rainfall gradient. Contrary to the biotic hypothesis, we found no evidence that leaf flush mediated arthropod biomass at landscape scales. This does not preclude changes in community composition on individual plants, and we also found taxa such as Orthoptera were more abundant on shrubs with new leaves, a result consistent with previous rainforest studies (Basset, 1991, 1996, 2001; Wardhaugh, 2014). In the wet tropics, chemical defense plays a role in reducing the palatability of new leaves (Coley & Barone, 1996; Gong et al., 2020), and we commonly observed delayed greening along with reddish coloration indicative of tannins and anthocyanins which could have reduced the importance of leaf flush in cloud forest. In evergreen tropical forest herbivores do not appear limited by food, whereas in seasonal dry forest and temperate regions deciduous leaves produce a dramatic change in resource availability (Dexter et al., 2018; Sanchez‐Azofeifa et al., 2005). At higher latitudes seasonal variation in photoperiod and temperature also contribute to greater synchronization among taxa (Thackeray et al., 2010).

Our results disentangle independent responses of plants and arthropods to abiotic drivers as we resolve a dynamic model for rainfall‐driven fluctuations in arthropod biomass. Unlike consistent biomass reductions in dry forest (Kishimoto‐Yamada & Itioka, 2015; Pinheiro et al., 2002; Tanaka & Tanaka, 1982), we documented extensive spatiotemporal variability. Many studies in wet systems have found that arthropods sometimes, but not always, decrease during the dry season (Boinski & Fowler, 1989; Buskirk & Buskirk, 1976; Fogden, 1972; Janzen, 1973; Janzen & Schoener, 1968; Valtonen et al., 2013; Wardhaugh et al., 2018). Here for the first time, we explain this variability by including nonlinearities, different time lags, and additive effects. Our biomass model is consistent with previous studies, but we show that arthropods increased rapidly in response to returning rain (1‐month accumulation) compared to greater time lags for arthropod decreases (3‐month accumulation). Rebounds after the dry season are consistent with 3–4 week time lags in drier systems (Tanaka & Tanaka, 1982) where insectivorous birds initiate nesting 3 weeks after returning rain to take advantage of abundant arthropod prey (Hidalgo Aranzamendi et al., 2019). Several studies have documented numerical changes in arthropods after 3 months (Kishimoto‐Yamada & Itioka, 2013; Nummelin & Nshubemuki, 1998; Valtonen et al., 2013) and across strata we found 90‐day rainfall accumulation in top models during the remainder of the year.

An important contribution from our research is the significance of intermediate rainfall to arthropods, which was evident in tropical montane cloud forest balanced between wet and dry extremes. Curvilinear relationships with rainfall were an unexpected result, and we found arthropods decreased not just after dry conditions (monthly rainfall <100 mm) but also after heavy rain (monthly rainfall >150 mm). In Costa Rican rainforest (annual rainfall >3000 mm) a single year of data showed drops in arthropod numbers during the rainy season in both foliage searches and sweep netting (Boinski & Fowler, 1989), and in similarly wet Australian rainforest, beetle diversity and numbers repeatedly peaked in October after several drier months (Grimbacher & Stork, 2009). In general, negative effects of heavy rain are poorly understood. Potential explanations for rainy season declines include direct effects of downpours on survival, development and foraging as shown experimentally for some caterpillars (Chen et al., 2019) and leaf‐cutter ants (Farji‐Brener et al., 2018). Additionally, near 100% daytime humidity could contribute to growth of entomopathogenic fungi (Hajek & St. Leger, 1994; Roy et al., 2005) commonly found in wet tropical forests (Evans, 1982).

In a humid system, we found compelling observational and experimental evidence for high drought susceptibility across cloud forest arthropod communities. Rainfall was likely the primary driver because arthropods maintain osmotic balance by drinking or obtaining water from their food (Danks, 2007; McCluney & Sabo, 2009). Evaporative drying magnified low rainfall, contributing to rapid biomass reductions during high solar insolation in the austral spring when VPD_max_ increased after three sunny days without rain (Newell et al., 2022c). Although we did not directly measure cloud water interception, moisture capture increases exponentially with RH (Prada et al., 2012), which could have increased soil water availability for plants. For arthropods, the importance of atmospheric moisture content was evident as additive effects of VPD_max_. Consistent with field data, our experiments showed poor desiccation resistance in most cloud forest taxa. Variability in the data indicated functional trait diversity with some species likely adapted to survive drier conditions. Intriguingly, color appeared linked to desiccation resistance in Lepidoptera larvae, primarily geometrids, which are especially diverse with 1000–2000 species across a single elevational gradient in the tropical Andes (Brehm et al., 2016). Several studies have documented production of melanin in drier climates to improve ‘waterproofing’ (Parkash et al., 2008; Välimäki et al., 2015), although lipid melting points are often discussed in the context of warming (Chown et al., 2011).

Climatic conditions influence arthropods on different time scales from shorter‐term changes in activity and behavior to longer‐term numerical changes. Several studies have found reduced activity of rainforest ants, beetles and termites during heavy rain (Basu, 1997; Dibog et al., 1998; Farji‐Brener et al., 2018; Grimbacher & Stork, 2009) and in Panamanian rainforest, aerial insects shifted activity from canopy gaps during the wet season to forest understory during the dry season (Richards & Windsor, 2007). High VPD can influence behavior (Becker et al., 2021), and in arid systems arthropods often seek moist refugia and enter dormancy (Denlinger, 1986). We found effects of rainfall were weaker for sampling methods with clear changes in activity such as greater numbers of aerial and leaf‐litter taxa during sunny, warm periods. Vigorous foliage branch beating appeared to dislodge even inactive arthropods, and during daytime sampling we commonly caught nocturnal Orthoptera (e.g. Gryllidae: Oecanthinae, Tettigoniidae). Although some herbivores could leave the plants where they forage, we rarely found green caterpillars or crickets in leaf litter. Thus, we interpret our results as primarily reflecting numerical changes although we cannot exclude changes in activity, especially for VPD_max_ with top models showing cumulative effects within days during dry conditions.

Strong interannual variability is consistent with rapid population dynamics on the scale of months. In the wet tropics, many taxa produce ≥6 overlapping generations, and generation times <30 days (Young, 2012) might explain numerical increases within a month compared to decreases over several generations. Further work is needed to understand mechanisms for community responses in our study. For example, populations of an Ethiopian butterfly peaked at intermediate rainfall because of decreased survival of young larvae and pupae during both heavy rain and dry conditions (Azerefegne et al., 2001). Another possibility is that survival and hatching of eggs depends on humidity for species without a protective membrane covering the egg (Jacobs et al., 2013). In Borneo rainforest, butterfly populations peaked at monthly rainfall between 100 and 200 mm, with reduced numbers after both drought and heavy rain (Hill et al., 2003). Across the lowland tropics, quarterly rainfall of 500–600 mm instead of 400 mm might be optimal for arthropods around 25°C based on temperature‐driven water stress (Thornthwaite, 1948). Further work is needed to apply our model across the lowland tropics, as well as refine ENSO‐related interannual variability and the role of evaporative drying during drought.

We show arthropod phenology shifted from wet‐to‐dry season maxima before plant communities transition to dry forest, typically characterized by seasonally deciduous trees with extended dry seasons >3 months (Dexter et al., 2018; Sanchez‐Azofeifa et al., 2005). In the Utcubamba and Marañon Valleys dry forests begin around 2000 m with annual rainfall <800 mm (e.g. Fabaceae‐dominated plant communities below the city of Chachapoyas). Rainfall isoclines where plant biomes transition contribute to abrupt community turnover (Gomez et al., 2020), and phenological shifts may be evidence for organisms adjusting to changing climate before reaching critical physiological or other thresholds. Disjuncts between arthropod and plant phenology imply that arthropods with short generation times respond to changes in rainfall before deep‐rooted plants influenced by soil water availability (Fang et al., 2021). Phenological responses to rainfall may differ from responses to warming as plants track extended growing seasons whereas consumers often lag behind warming (Thackeray et al., 2010).

Rapid response of arthropods to rainfall extremes could have cascading effects on consumers. Invertebrates contribute at least half of the diet for 57% of bird and 35% of mammal species (Wilman et al., 2014) and >60% of Neotropical bird species are considered insectivores (Sherry, 2021). Increasing environmental stochasticity amplifies synchronized population fluctuations (Yang et al., 2019) while rapid directional climate change drives population trends (Spooner et al., 2018). Our arthropod biomass model provides a testable hypothesis for insectivore declines in both wet and dry regions (Figure 5c) if the ‘wet‐get‐wetter and dry‐get‐drier’ (Held & Soden, 2006). With reduced rainfall in wet regions and greater rainfall in dry regions, insectivore populations might increase with population size contributing to competition and density‐dependent feedback mechanisms (Ferguson & Ponciano, 2015). Alternatively, dietary specialists might respond to changes in specific invertebrate taxa. Experts warn of fewer arthropods (Janzen & Hallwachs, 2021), but lack of long‐term data makes tropical trends difficult to assess (Sánchez‐Bayo & Wyckhuys, 2019; van Klink et al., 2020). In Costa Rica, 22‐year declines in caterpillar and parasitoid richness were linked to increasing temperature and rainfall anomalies (Salcido et al., 2020) although moth populations in Ecuador appear stable (Wagner et al., 2021). Tropical birds have received greater study, and 40 years of data from Panamanian rainforest show dry season length drives demographic variability (Brawn et al., 2017), while a number of studies have documented enigmatic declines in undisturbed tropical rainforest, especially for understory or terrestrial insectivores likely linked to climate change (Blake & Loiselle, 2015; Curtis et al., 2021; Pollock et al., 2022; Sigel et al., 2006; Stouffer et al., 2021).

In general, our results have broad application for understanding responses to climate change across different taxa and trophic groups (Thackeray et al., 2010). Concurrent insect‐insectivore declines might serve as an early warning for longer‐term consequences of changing rainfall in the tropics. For ‘the little things that run the world’ (Wilson, 1987), decreasing biomass and abundance of invertebrates under both wet and dry extremes could have cascading effects on a range of ecosystem functions from pollination to decomposition depending on how individual species and functional groups are affected. Our results show rapid response of arthropods to changing rainfall regimes, but funding for longer‐term monitoring is needed to understand how short‐term population fluctuations influence long‐term population trends as density‐dependence and adaption play important roles in population dynamics over time (Didham et al., 2020). Given the critical importance of invertebrates and the potential for climate change to alter food webs and destabilize ecological function, global policy changes are urgently needed to reduce emissions and protect some of the world's most biodiverse ecosystems before they are further impacted by climate change and other anthropogenic disturbances.

## AUTHOR CONTRIBUTIONS

Felicity L. Newell conceived and designed the study with advice from Scott K. Robinson. Felicity L. Newell and Ian J. Ausprey collected the data and all authors contributed funding. Felicity L. Newell analyzed the data and wrote the manuscript with significant contributions from an Ian J. Ausprey and Scott K. Robinson.

## CONFLICTS OF INTEREST

The authors declare that there is no conflict of interest.

## Supporting information


Appendix S1

## Data Availability

The data that support the findings of this study are available on Figshare. Weather data from Newell et al. (2022a) at http://doi.org/10.6084/m9.figshare.1854316. Arthropod datasets and scripts used to generate figures from Newell et al. (2022b) at http://doi.org/10.6084/m9.figshare.19107188.
