# Supplementary material for: Wet and dry extremes reduce arthropod biomass independently of leaf phenology in the wet tropics
Source: Glob Chang Biol. 2022 Sep 14;29(2):308–23. doi: 10.1111/gcb.16379 (PMC10087840; doi:10.1111/gcb.16379)
Supplement: Supplementary file 1 — Appendix S1 [file GCB-29-308-s001.pdf]

Supporting Information for:

Wet and dry extremes reduce arthropod biomass independently of leaf phenology in the wet tropics

Felicity L. Newell, Ian J. Ausprey, and Scott K. Robinson

Email: fnewell@ufl.edu, felicity.newell@gmail.com

This PDF file includes:

SI Methods…………………………………......................... Pages 2–3

SI Results……………………………….............................Page 3

SI References………………………………………………...... Page 4

SI Figures S1 to S9………………………………………….. Pages 5–13

SI Tables S1 to S8…………………………………………....Pages 14–21

SI Desiccation resistance experiments…………………… Pages 22–26
Figure S10, Tables S9 to S10

SI Arthropod biomass model……………………………...Pages 27–30
Figures S11 to S14

SI Project collaborators in Perú……………………………Pages 31–32

SI Methods

Weather data.
For cloud cover, we calculated the percentage of pixels occupied by cloud within a 5-km radius at a 250 m resolution from MOD09GA (https://lpdaac.usgs.gov/products/mod09gav061/). We recorded forest microclimate inside the forest using Hobowear data loggers (U23-001) located in a dense closed-canopy area at a standard height of 1.5 m (Newell *et al.* 2022). We calculated atmospheric pressure from temperature and elevation, and vapor pressure deficit (VPD) from temperature and relative humidity (RH) using the package *psychrolib* (Meyer & Thevenard 2019). We used the normal ratio method (Paulhus & Kohler 1952; Mair & Fares 2010) to scale rainfall from long-term weather stations in the region (SENAMHI 2020) to local landscapes based on *in situ* gauge data (Newell *et al.* 2022). To examine different climatic time lags, we used rolling means and sums using the package *data.table* (Dowle & Srinivasan 2020) and an initial model selection analysis to select the time lag that best fit the data.

Satellite vegetation greenness.
We downloaded 16-day 250-m resolution MODIS EVI grids for northern Peru using the MODISstp package (Busetto & Ranghetti 2016) from the USGS and NASA EarthData LP DAAC website (https://lpdaac.usgs.gov/products/mod13q1v006/). We extracted quality assessment bitcode for each pixel using the MODIS package (Mattiuzzi & Detsch 2020). Pixels were filtered to remove cloud cover or other obstructions using the following data filters (Samanta *et al.* 2010): QA <2, usef <13, mix_cld=0, adj_cld=0, shd=0, aer%in%c(1,2). We selected pixels within a 300-m elevation band of our study sites and filtered for >50% forest cover using global data averaged for each 250-m grid cell (Hansen *et al.* 2013). For each landscape we used 6-wk running medians weighted by usefulness to smooth EVI data calculated using the Hmisc package (Harrell *et al.* 2020).

Arthropod transects.
Transects at each site were located along mist-net lanes, which for logistical reasons were either (a) linearly placed along trails generally parallel to the slope or (b) in a circular loop. Observers walked transects in opposite directions along mist-net arrays or at different sites, and we used pacing to locate points at 30-m intervals. During sampling we counted and collected all visible invertebrates ≥ 1mm in length; individuals were stored in ethanol by visit for further identification and analysis as needed. Calipers were used to calibrate size estimation which facilitated rapid counting of invertebrates. Arthropod sampling was conducted from 9am to 6pm, with the most common sampling time in early afternoon (Figure S4). We occasionally sampled during light drizzle but avoided heavy rain and recorded when vegetation was wet. For all sampling, we used a collapsible 30-cm diameter pocket-net and 74-cm sweep-net pole which we also used to calibrate the sampling area (BioQuip 7112CP and 7312AA).

Foliage branch beating.
To randomly sample understory plants, at each point we selected the nearest vegetation 1–2 m in height with ≥50% foliage. Instead of using a tray or sheet, we vigorously beat a 0.5 m^2 area of foliage several times over a sweep-net held underneath the branch which we rapidly closed upon completion to capture jumping or flying insects.

Aerial sweep-netting.
Sweep netting was modified to sample primarily aerial insects, and at each point we conducted 12 sweeps approximately 1-m apart in the nearest relatively open area such as along mist-net lanes (Murphy 1986; Cooper & Whitmore 1990). We rapidly closed the net upon completion to stop jumping or flying insects from escaping, carefully opening sections to first collect active individuals.

Leaf-litter quadrants.
We counted invertebrates in the nearest undisturbed area with ≥50% leaf-litter (excluding bare ground and herbaceous vegetation). At each point we sampled a 0.5 m^2 quadrant. After first counting any active jumping or flying insects, we spent around 5 min flipping over leaves searching for invertebrates in the top layer of loose organic material (Strong & Sherry 2000; Diggs *et al.* 2011).

Biomass calculations.
To calculate dry mass we used length × width equations with the largest sample size from the humid tropics (Gruner 2003; Wardhaugh 2013) or length equations from other published studies primarily from the tropics (Collins 1992; Ganihar 1997; Johnson 2000) (Table S1). To calculate total biomass, we dropped exceptionally large cloud forest taxa which skewed biomass including Gastropoda, Myriapoda and a few adult Odonata found in July and Oct 2017, likely migrating across the highest elevation site. For the analysis by sample, extreme outliers (1–3 per analysis driven by clusters or an unusually large individual) were examined visually and replaced by trimmed maxima (winsorizing). Temporal variability proved to be the strongest factor, and for the analysis by landscape-visit data were pooled across sub-sites (less variability), observers (stratified by visit), and plant species (sampled as available).

SI Results

Aerial arthropods.
Consistent with foliage arthropods, 90-day rainfall accumulation was in the top model for aerial insects (Table S6). Rain affected activity of aerial insects in the short term, and biomass increased 4% per dry day since rain while biomass was 26% lower when vegetation was wet. However, on longer time scales insect biomass from sweep nets decreased as the system dried out with a 35% reduction per 300 mm less rain in the previous 90-days (Figure S7a); the top additive model with days since rain plus 90-day rainfall accumulation explained 29% of the variation. Short-term responses to rainfall were significant for most aerial taxa: Diptera, Lepidoptera adults, and Araneae, while effects of drying out were only apparent for Hymenoptera (Figure S9).

Leaf-litter arthropods.
Like other methods, 90-day rainfall accumulation was also in the top model for leaf-litter invertebrates (Table S7). Activity of leaf-litter invertebrates responded weakly to temperature in the short-term with slightly greater biomass from samples collected on warm days. Additionally, biomass was 19% higher for afternoon compared to morning samples. Evidence for numerical changes, arthropod biomass showed a weak curvilinear response to 90-day rainfall accumulation with similar peaks as foliage arthropods around 130 mm^{-1} for three months (Figure S7b); the top model explained 21% of the variation. None of the models explained biomass of leaf-litter invertebrates when including Gastropoda and Myriapoda, and for individual leaf-litter taxa rainfall was not significant.

SI References

Busetto, L. & Ranghetti, L. (2016). MODIStsp: an R package for preprocessing of MODIS Land Products time series. *Computers & Geosciences*, 97, 40–48.

Collins, P.T. (1992). Length-biomass relationships for terrestrial Gastropoda and Oligochaeta. *American Midland Naturalist*, 128, 404.

Cooper, R. & Whitmore, R.C. (1990). Arthropod sampling methods in ornithology. *Stud. Avian Biol.*, 13, 29–37.

Diggs, N.E., Marra, P.P. & Cooper, R.J. (2011). Resource limitation drives patterns of habitat occupancy during the nonbreeding season for an omnivorous songbird. *The Condor*, 113, 646–654.

Dowle, M. & Srinivasan, A. (2020). *data.table: Extension of `data.frame`. R package version 1.13.0.*

Ganihar, S.R. (1997). Biomass estimates of terrestrial arthropods based on body length. *Journal of Biosciences*, 22, 219–224.

Gruner, D.S. (2003). Regressions of length and width to predict arthropod biomass in the Hawaiian Islands. *Pacific Science*, 57, 325–336.

Hansen, M.C., Potapov, P.V., Moore, R., Hancher, M., Turubanova, S.A., Tyukavina, A., *et al.* (2013). High-Resolution Global Maps of 21st-Century Forest Cover Change. *Science*, 342, 850–853.

Harrell, F.E.Jr., Dupont, with contributions from C. & others, many. (2020). *Hmisc: Harrell Miscellaneous*.

Johnson, M.D. (2000). Evaluation of an arthropod sampling technique for measuring food availability for forest insectivorous birds. *Journal of Field Ornithology*, 71, 88–109.

Lefcheck, J.S. (2016). piecewiseSEM: Piecewise structural equation modelling in R for ecology, evolution, and systematics. *Methods in Ecology and Evolution*, 7, 573–579.

Mair, A. & Fares, A. (2010). Assessing rainfall data homogeneity and estimating missing records in Mākaha Valley, Oʻahu, Hawaiʻi. *Journal of Hydrologic Engineering*, 15, 61–66.

Mattiuzzi, M. & Detsch, F. (2020). *MODIS: Acquisition and processing of MODIS products. R package version 1.2.2*.

Meyer, D. & Thevenard, D. (2019). PsychroLib: a library of psychrometric functions to calculate thermodynamic properties of air. *Journal of Open Source Software*, 4, 1137.

Murphy, M.T. (1986). Temporal components of reproductive variability in Eastern Kingbirds (Tyrannus tyrannus). *Ecology*, 67, 1483–1492.

Newell, F.L., Ausprey, I.J. & Robinson, S.K. (2022). Spatiotemporal climate variability in the Andes of northern Peru: Evaluation of gridded datasets to describe cloud forest microclimate and local rainfall. *Int. J. Climatol.*, n/a.

Paulhus, J.L. & Kohler, M.A. (1952). Interpolation of missing precipitation records. *Monthly Weather Review*, 80, 129–133.

Samanta, A., Ganguly, S., Hashimoto, H., Devadiga, S., Vermote, E., Knyazikhin, Y., *et al.* (2010). Amazon forests did not green-up during the 2005 drought. *Geophysical Research Letters*, 37.

Strong, A.M. & Sherry, T.W. (2000). Habitat-specific effects of food abundance on the condition of Ovenbirds wintering in Jamaica. *J. Anim. Ecol.*, 69, 883–895.

Wardhaugh, C.W. (2013). Estimation of biomass from body length and width for tropical rainforest canopy invertebrates: Invertebrate biomass measures. *Australian Journal of Entomology*, 52, 291–298.

SI Figures

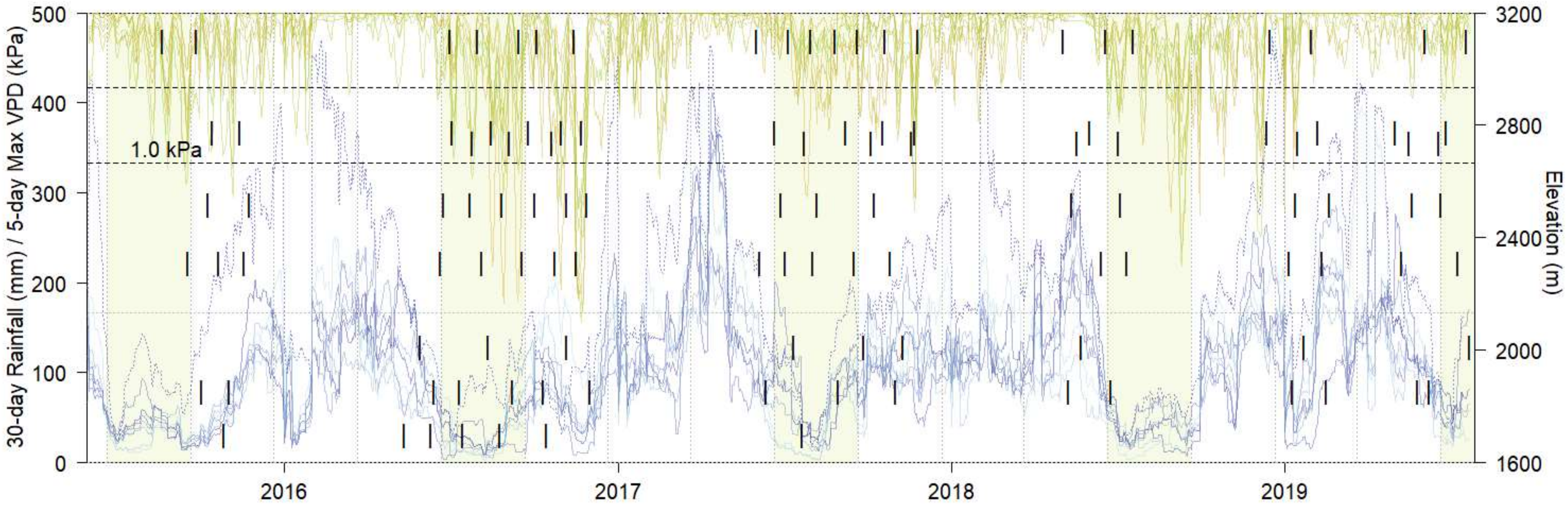

Figure S1. Arthropod sampling design across years and elevations in relation to spatiotemporal weather variation. Sampling visits (black bars) captured five years of variation in local rainfall (blue lines, left y-axis) and vapor pressure deficit (VPD) (yellow lines, dashed black lines indicate water stress thresholds) across a 1700–3100 m elevational gradient (right y-axis). Shaded areas indicate austral winter (dry season). Cloud forest weather across elevations at 5–7°S in the Andes of northern Peru adapted from Newell et al. (2022c).

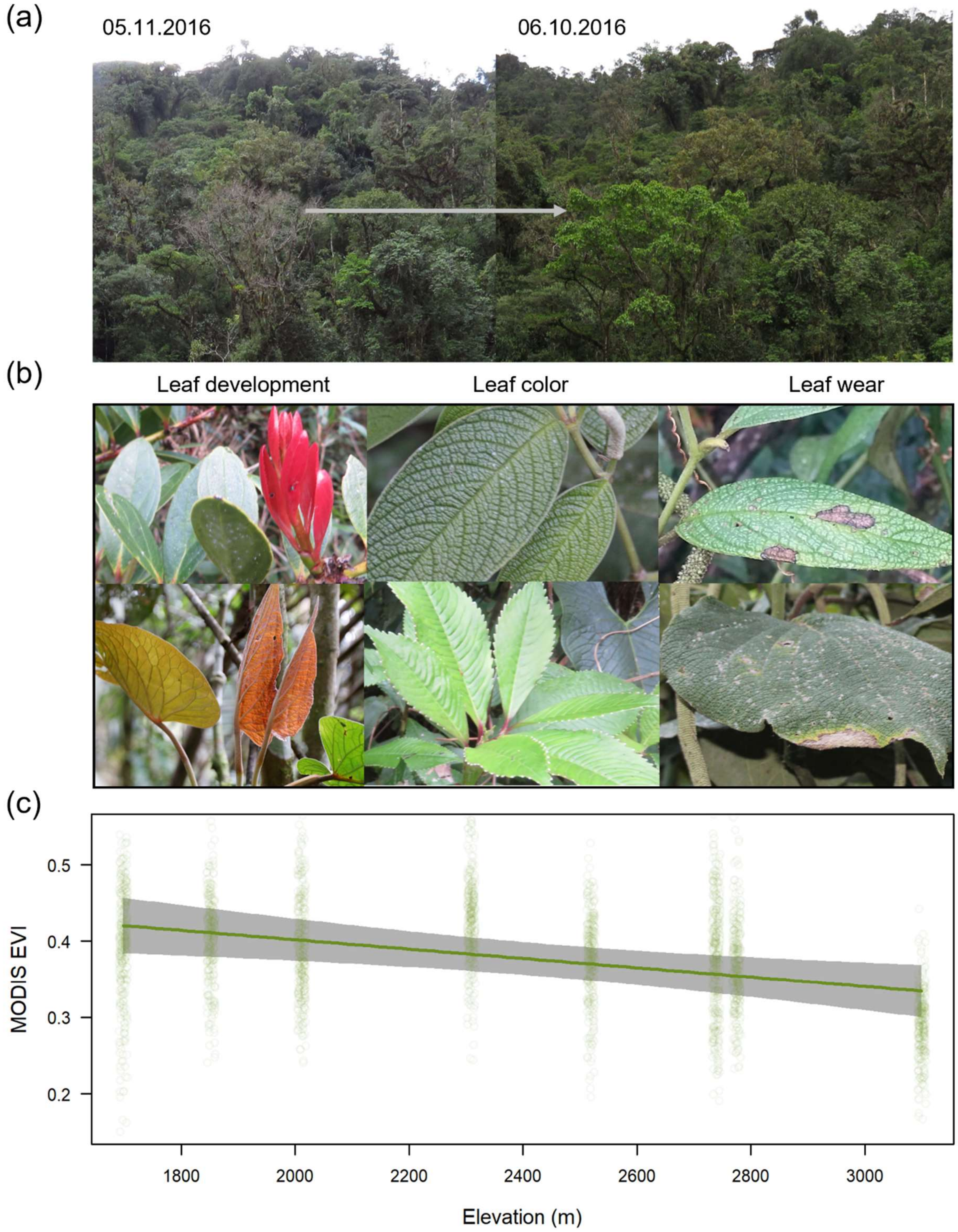

Figure S2. *In situ* leaf phenology was recorded for 100 canopy trees and 100 understory shrubs during each landscape visit. (a) New leaves on were characterized by bright green coloration after leaf flush and canopy trees were scored in the field through binoculars, as well as documented with repeat photographs from canopy vantage points. (b) A combination of developing leaves, bright green coloration, and leaf wear were used to classify plants as >33% initiating, new or old leaves. (c) The enhanced vegetation index (EVI) from the MODIS Terra satellite decreased with elevation. Photo credits F.L. Newell.

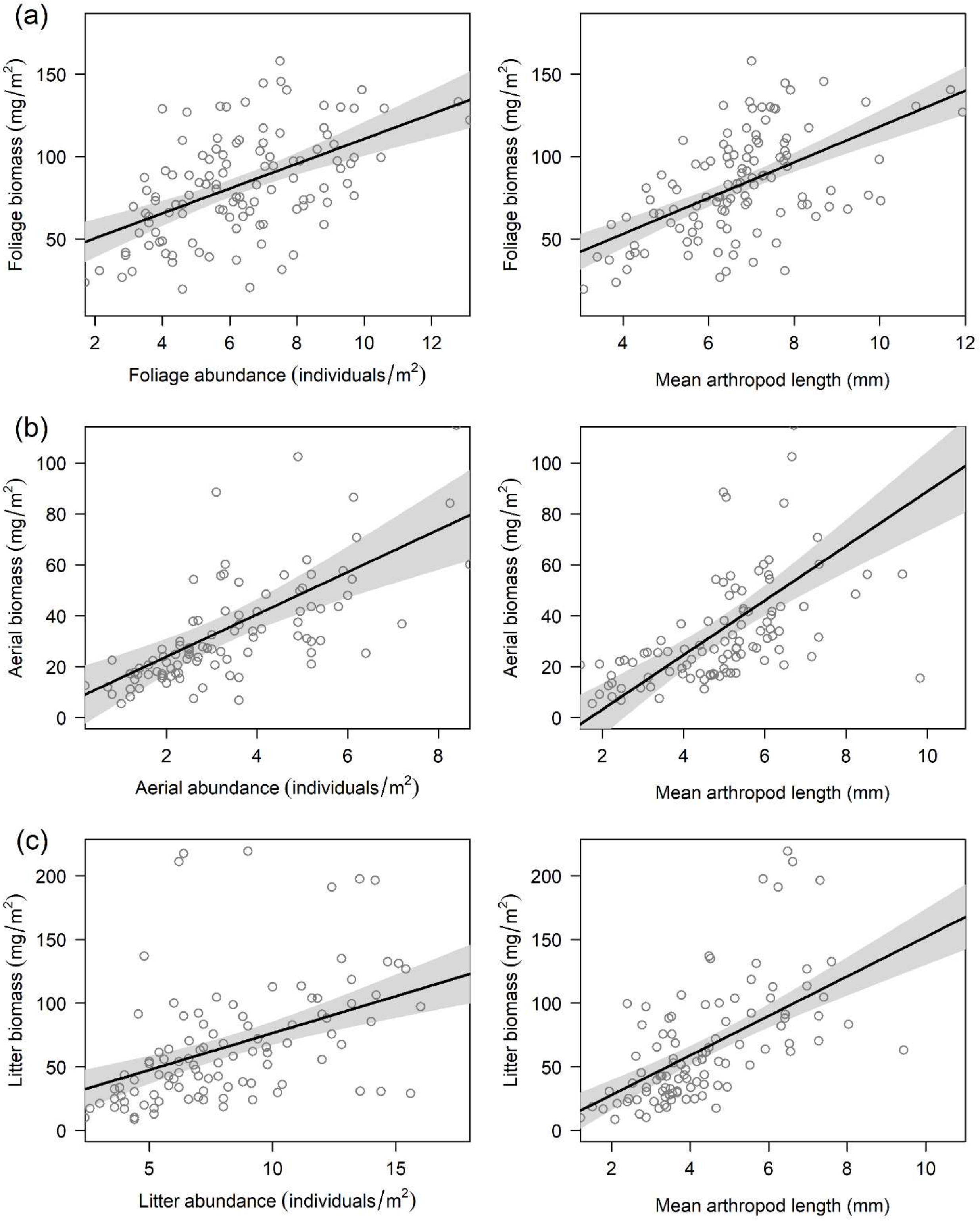

Figure S3. Relationship between arthropod biomass and abundance for individuals > 3 mm in length from (a) foliage branch beating, (b) aerial insects, and (c) leaf-litter quadrants. Points represent > 100 visits across a network of 8 cloud forest landscapes in northern Peru, 2015–2019. A few single outliers not shown on graph.

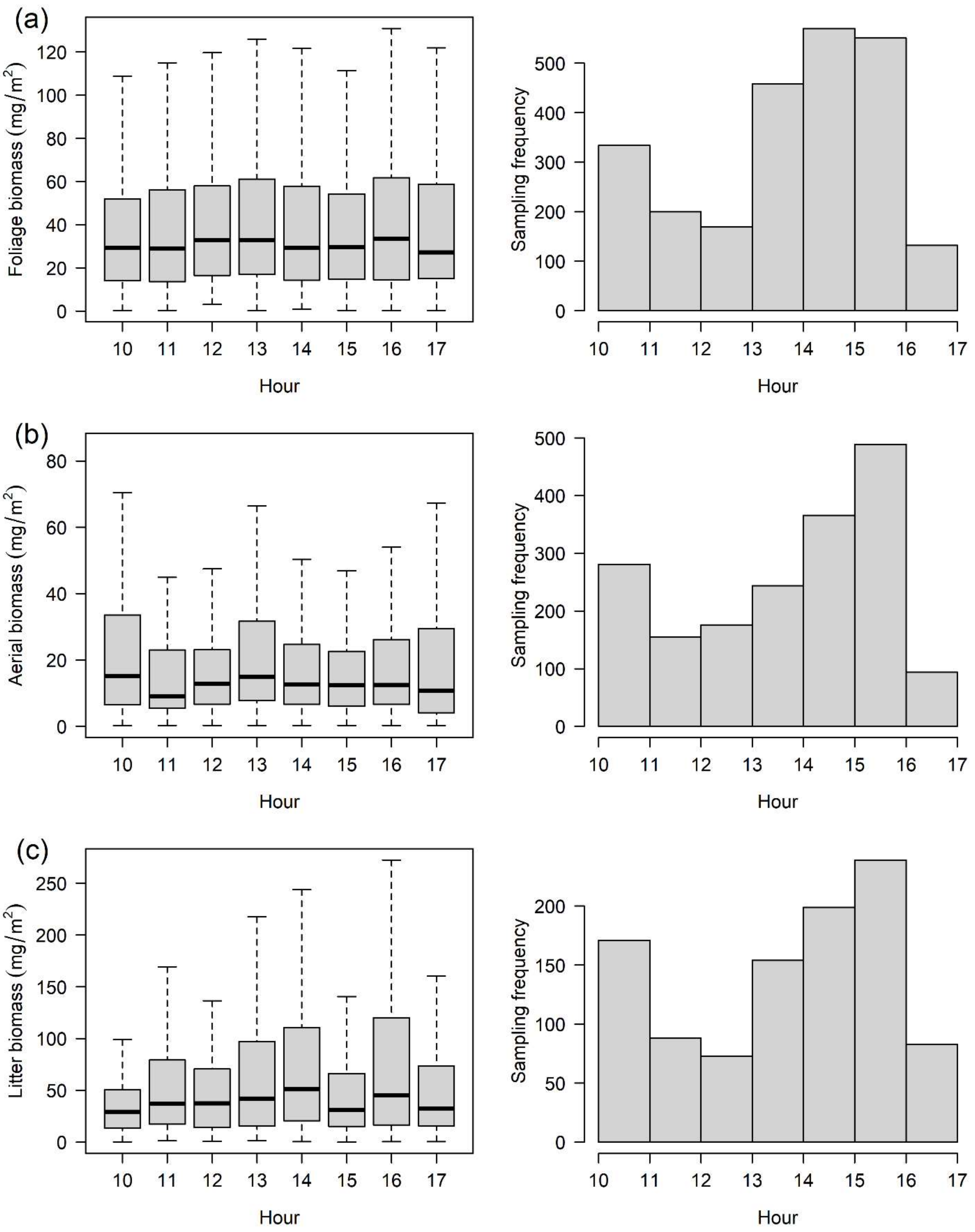

Figure S4. Arthropod biomass and sampling effort by time of day for (a) foliage branch-beating, (b) aerial sweep-nets, and (c) leaf-litter quadrants. Sampling across a network of cloud forest sites in the Chachapoyas region of northern Peru (116 visits, 8 landscapes, 2015–2019).

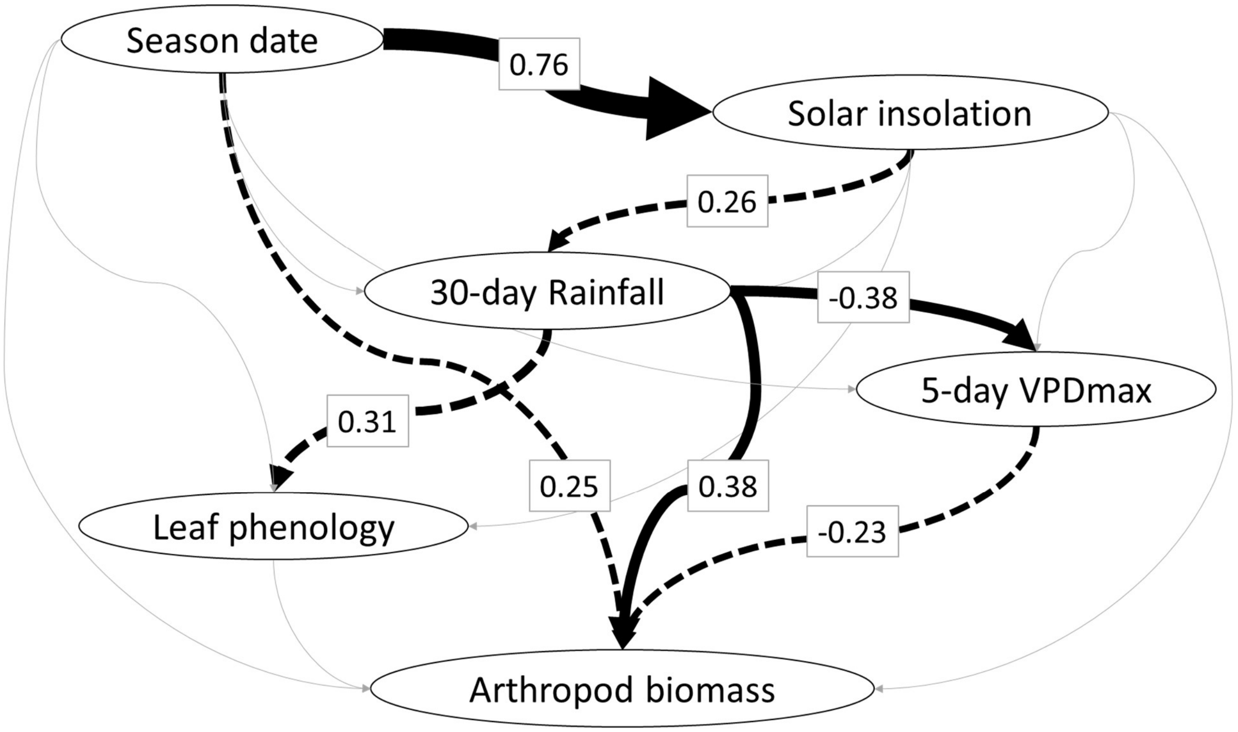

Predictor	Response	beta	SE	df	T_{crit}	*P*-val	Std.Estimate
Season date	Solar insolation	0.312	0.035	50	74.9	0.000	0.76
Season date	30-day Rainfall	0.213	0.121	28	2.4	0.135	0.27
Solar insolation	30-day Rainfall	0.497	0.239	12	3.3	0.096	0.26
Season date	5-day VPDmax	0.002	0.001	21	1.1	0.308	0.22
Solar insolation	5-day VPDmax	0.004	0.003	20	0.8	0.382	0.18
30-day Rainfall	5-day VPDmax	-0.004	0.002	52	5.6	0.021	-0.38
Season date	Leaf phenology	0.001	0.001	9	1.9	0.201	0.20
Solar insolation	Leaf phenology	0.001	0.001	28	0.7	0.404	0.15
30-day Rainfall	Leaf phenology	0.002	0.001	17	3.4	0.082	0.31
5-day VPDmax	Leaf phenology	-0.001	0.058	39	0.0	0.988	0.00
Leaf phenology	Arthropod biomass	23.359	144.355	51	0.0	0.879	0.02
Season date	Arthropod biomass	1.079	0.491	9	3.9	0.081	0.25
Solar insolation	Arthropod biomass	-0.695	1.530	26	0.2	0.689	-0.07
30-day Rainfall	Arthropod biomass	2.018	0.677	20	6.1	0.022	0.38
5-day VPDmax	Arthropod biomass	-123.248	59.131	31	3.0	0.092	-0.23

Figure S5. Structural equation modeling for periods of increasing rainfall after the dry season. (a) Solid lines indicate significant variables ($P \leq 0.05$), dashed lines a tendency ($P \leq 0.10$), and gray lines were not significant. (b) Coefficients standardized by scaling standard deviations using the package piecewiseSEM (Lefcheck 2016). Random effects included a crossed random space × time intercept with fit approximated using a gaussian distribution. We were not able to show non-linear relationships at other times of year due to limitations with the SEM approach.

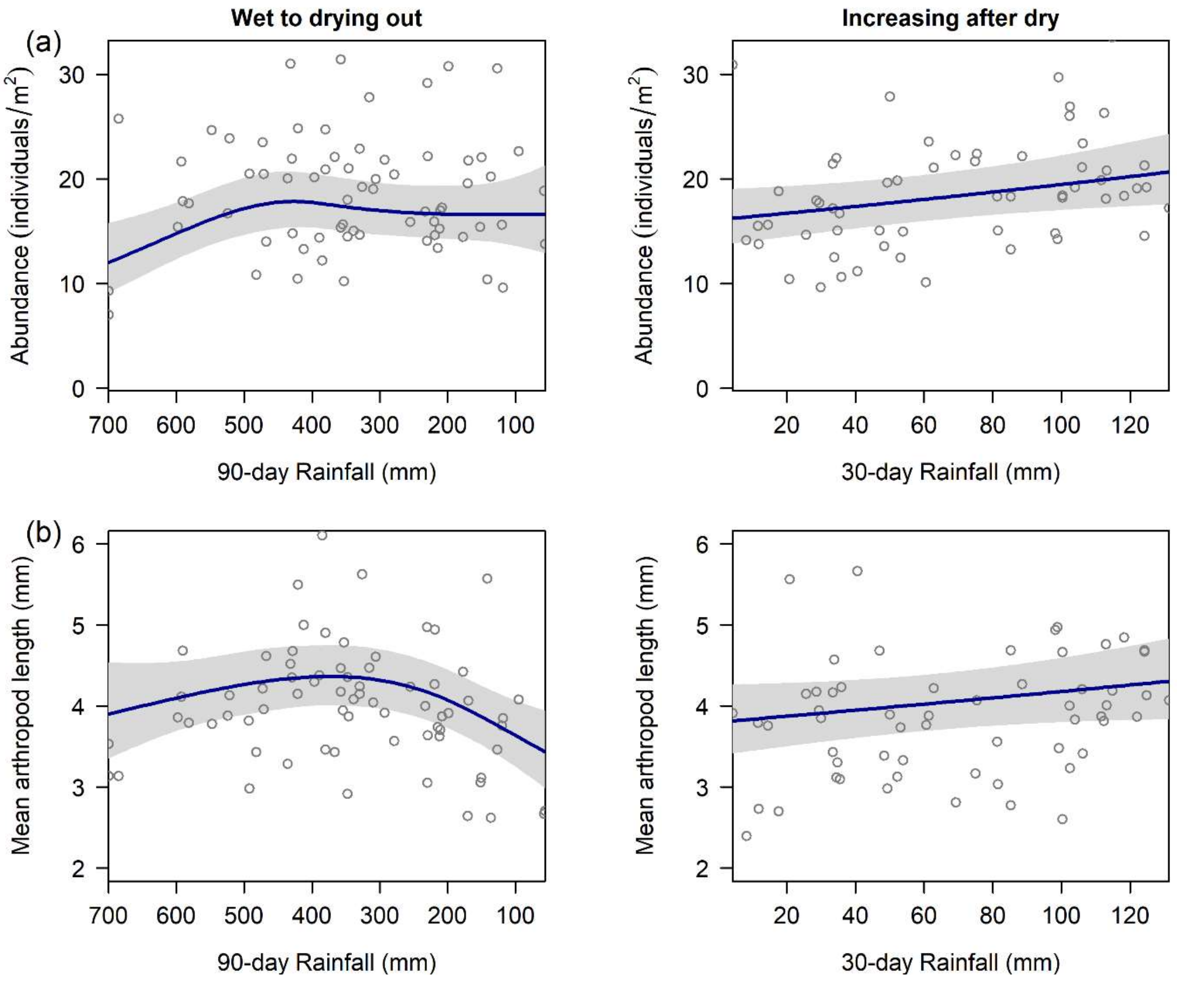

Figure S6. Different components of arthropod biomass relative to rainfall accumulation time lags pre (70 visits) and post the dry season (58 visits, monthly rainfall <130 mm). (a) Arthropod abundance and (b) mean length for foliage branch beating across a network of cloud forest sites in the Chachapoyas region of northern Peru (116 visits, 8 landscapes, 2015–2019). Gray polygons represent 95% CI.

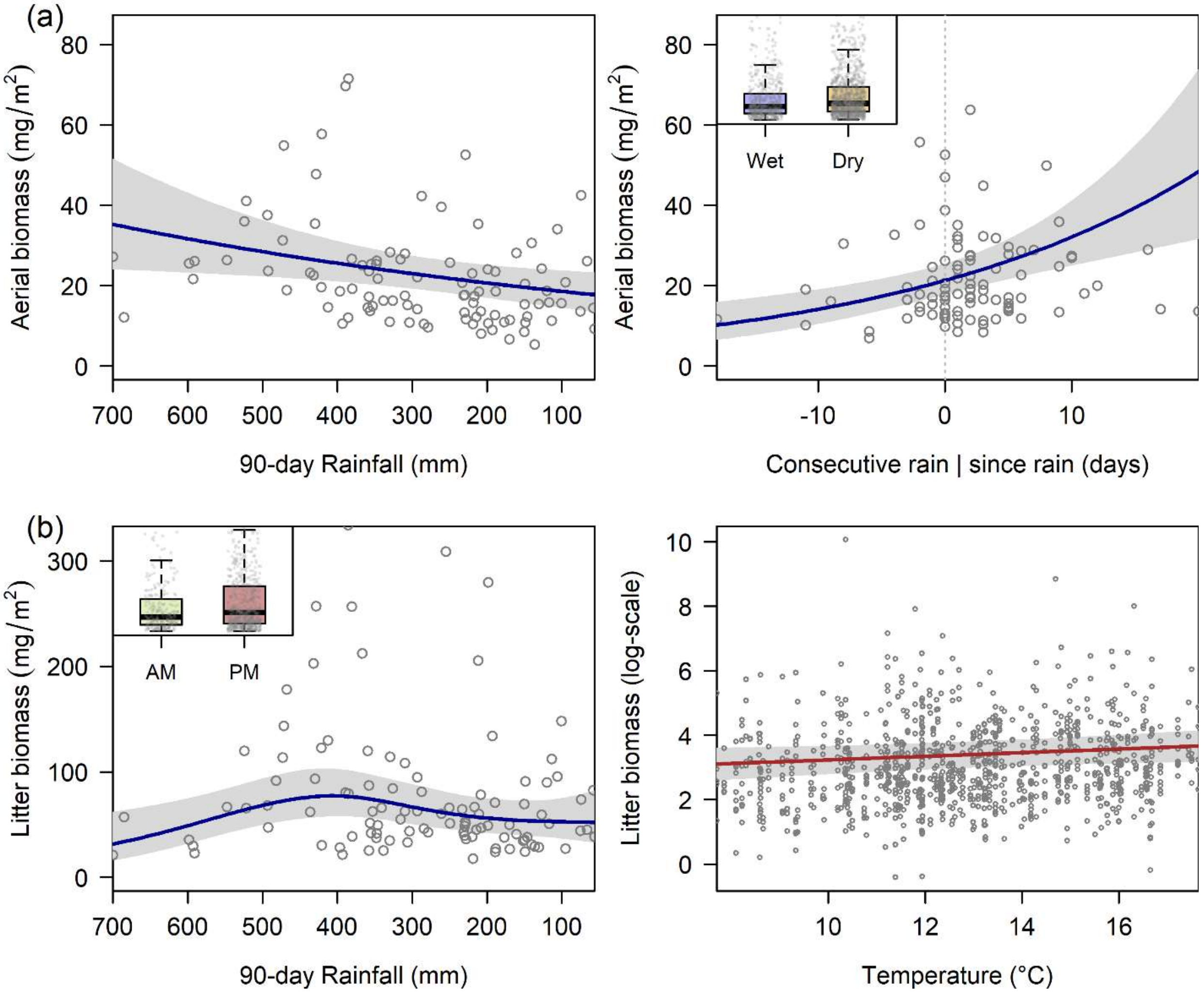

Figure S7. Top models for spatiotemporal variation in arthropod biomass using other sampling methods. Partial residual plots of response to rainfall for (a) aerial insects and (b) leaf-litter invertebrates. Insets and temperature represent top models at the scale of the sample showing short-term changes in activity. Aerial sweep netting and leaf-litter quadrants from a network of cloud forest sites in the Chachapoyas region of northern Peru (>100 visits, 8 landscapes, 2016–2019). Gray polygons represent 95% confidence intervals.

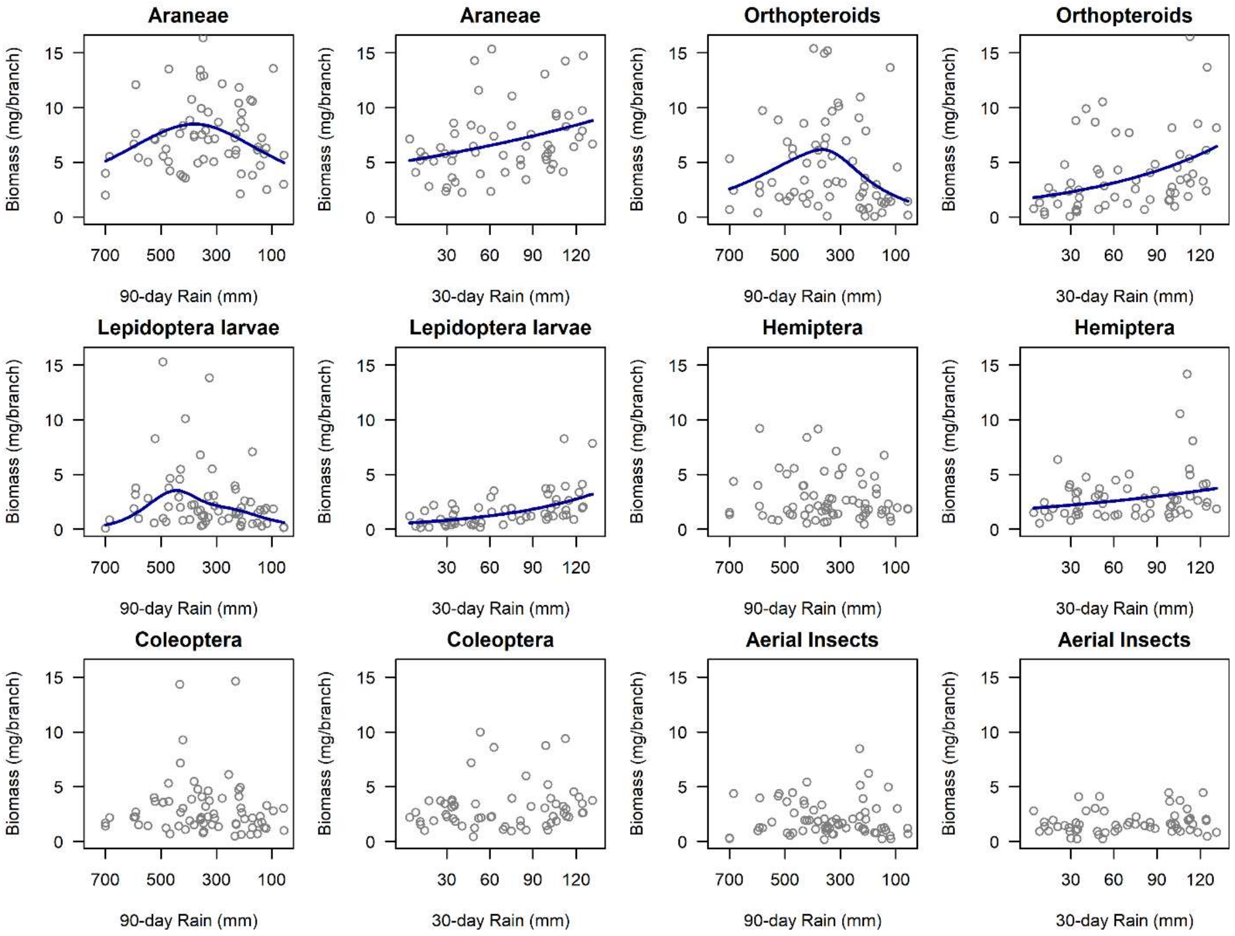

Figure S8. Response of common orders of foliage arthropods to rainfall pre/post the dry season. Orthopteroids include the orders Orthoptera + Phasmida, and aerial insects include the orders Diptera + Hymenoptera + adult Lepidoptera. Regression lines shown for significant trends ($P < 0.05$).

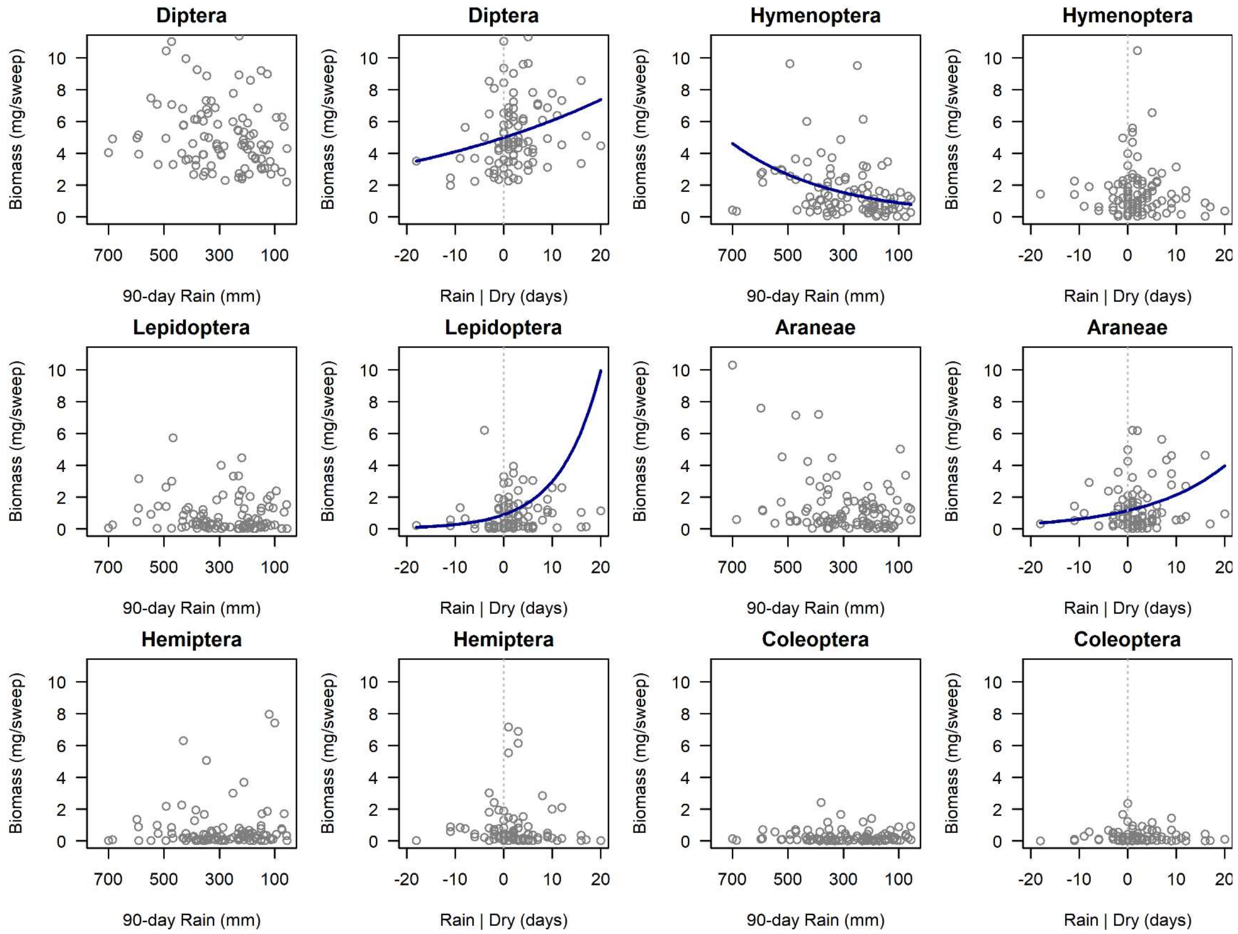

Figure S9. Response of common orders of aerial insects to rainfall. Lepidoptera includes larvae and adults. Regression lines shown for significant trends (*P* < 0.05).

SI Tables

Table S1. Regression coefficients used to calculate arthropod biomass by taxa. Dry mass (mg) was calculated using either length × width (LW) or length (L) equations for humid tropical systems depending on availability of published coefficients.

Order	*n*	*a*	*b*	Equation	Citation
Gastropoda	320	-2.75	1.59	$e^{a}LW^{b}$	Wardhaugh 2013
Lumbricidae	64	-11.90	2.29	$e^{a}L^{\mathbf{b}}$	Collins 1992
Chilopoda	443	-6.70	2.84	$e^{a}L^{\mathbf{b}}$	Ganihar 1997
Diplopoda	285	-6.70	2.84	$e^{a}L^{\mathbf{b}}$	Ganihar 1997
Opiliones	305	-2.88	2.33	$e^{a}L^{\mathbf{b}}$	Gruner 2003
Acari	353	-2.88	2.33	$e^{a}L^{\mathbf{b}}$	Gruner 2003
Araneae	7575	-0.91	1.18	$e^{a}LW^{b}$	Wardhaugh 2013
Isopoda	617	-2.94	1.49	$e^{a}LW^{b}$	Wardhaugh 2013
Collembola	483	-1.87	2.30	$e^{a}L^{\mathbf{b}}$	Ganihar 1997
Thysanura	143	-2.59	1.67	$e^{a}L^{\mathbf{b}}$	Ganihar 1997
Dermaptera	335	-5.65	2.49	$e^{a}L^{\mathbf{b}}$	Johnson 2000
Orthoptera	1359	-1.34	1.29	$e^{a}LW^{b}$	Wardhaugh 2013
Blattodea	1408	-2.80	1.41	$e^{a}LW^{b}$	Wardhaugh 2013
Thysanoptera	489	-3.55	1.25	$e^{a}LW^{b}$	Wardhaugh 2013
Phasmida	194	-2.52	1.10	$e^{a}LW^{b}$	Wardhaugh 2013
Hemiptera	3094	-1.71	1.21	$e^{a}LW^{b}$	Wardhaugh 2013
Psocoptera	497	-4.30	3.12	$e^{a}L^{\mathbf{b}}$	Gruner 2003
Hymenoptera	1858	-2.10	1.34	$e^{a}LW^{b}$	Wardhaugh 2013
Formicidae	153	-3.11	1.61	$e^{a}LW^{b}$	Wardhaugh 2013
Neuroptera	82	-4.96	2.74	$e^{a}L^{\mathbf{b}}$	Gruner 2003
Coleoptera	4053	-2.32	1.38	$e^{a}LW^{b}$	Wardhaugh 2013
Lepidoptera (adult)	417	-4.79	2.86	$e^{a}L^{\mathbf{b}}$	Ganihar 1997
Lepidoptera (larvae)	1723	-2.85	1.30	$e^{a}LW^{b}$	Wardhaugh 2013
Diptera	8835	-1.91	1.22	$e^{a}LW^{b}$	Wardhaugh 2013

Table S2. Summary of arthropod taxa collected from three different cloud forest strata. Counts, mean length ± SE, %total, and %biomass for all individuals by strata (foliage: *n* = 22189, aerial: *n*= 17255, and leaf-litter: *n* = 8653) from the Chachapoyas region of northern Peru, 2015-2019.

			Foliage Branch-Beatings							Aerial Sweep Nets							Leaf-litter Quadrants						
		Taxa	#	Length (mm)			%T	%B	#	Length (mm)			%T	%B	#	Length (mm)			%T	%B
		Mollusca: Gastropoda	94	4.3	±	0.5	0.4	3.3	3	3.3	±	1.9	0.0	0.0	239	8.3	±	0.5	2.8	8.8
		Annelida: Lumbricidae	2	5.0	±	1.0	0.0	0.0	-	-	-	-	-	-	111	20.1	±	2.6	1.3	5.2
		Myriapoda: Chilopoda	74	7.1	±	0.4	0.3	0.1	-	-	-	-	-	-	392	9.8	±	0.3	4.5	1.9
		Diplopoda	43	7.0	±	0.6	0.2	0.0	-	-	-	-	-	-	268	15.3	±	0.7	3.1	4.9
		Arachnida: Opiliones	249	3.7	±	0.1	1.1	0.8	34	2.3	±	0.3	0.2	0.1	55	4.4	±	0.4	0.6	0.4
		Acari	339	1.3	±	0.0	1.5	0.1	16	1.6	±	0.2	0.1	0.0	70	1.2	±	0.1	0.8	0.0
		Araneae	6275	2.7	±	0.0	28.3	31.1	633	2.9	±	0.1	3.7	11.6	1301	3.3	±	0.1	15.0	20.3
		Crustacea: Isopoda	119	4.3	±	0.2	0.5	0.4	8	4.5	±	0.6	0.0	0.1	632	4.5	±	0.1	7.3	3.8
		Hexapoda:Collembola	130	2.1	±	0.1	0.6	0.2	-	-	-	-	-	-	670	2.0	±	0.0	7.7	1.3
		Archaeognatha[1]	86	5.4	±	0.4	0.4	0.2	16	4.2	±	0.5	0.1	0.1	64	4.3	±	0.3	0.7	0.1
		Dermaptera	191	6.8	±	0.3	0.9	0.2	13	4.5	±	0.9	0.1	0.0	177	7.6	±	0.5	2.0	0.5
		Orthoptera	856	6.0	±	0.1	3.9	17.6	42	3.9	±	0.5	0.2	1.0	544	6.0	±	0.2	6.3	16.3
Arthropoda		Blattodea	399	4.3	±	0.2	1.8	1.5	5	5.2	±	1.2	0.0	0.1	1299	6.3	±	0.1	15.0	17.7
		Isoptera	2	3.5	±	0.1	0.0	0.0	1	3.6	±	0.2	0.0	0.0	30	3.5	±	0.2	0.3	0.2
		Thysanoptera	397	3.9	±	0.1	1.8	0.2	48	3.7	±	0.3	0.3	0.0	148	4.1	±	0.2	1.7	0.1
		Phasmida	158	17.6	±	0.7	0.7	1.3	13	17.3	±	1.9	0.1	0.3	23	18.3	±	3.5	0.3	0.4
	Insecta	Hemiptera	2692	3.9	±	0.0	12.1	11.3	409	3.8	±	0.1	2.4	4.7	442	3.3	±	0.1	5.1	2.1
		Psocodea	381	3.6	±	0.1	1.7	1.1	158	3.5	±	0.1	0.9	1.3	16	3.4	±	0.5	0.2	0.1
		Hymenoptera	697	3.5	±	0.1	3.1	1.6	1864	3.9	±	0.1	10.8	14.7	167	4.4	±	0.4	1.9	0.8
		Formicidae	70	3.2	±	0.4	0.3	0.1	5	4.0	±	2.0	0.0	0.0	188	3.8	±	0.4	2.2	0.5
		Neuroptera	69	3.5	±	0.1	0.3	0.5	18	3.6	±	0.2	0.1	0.3	5	3.5	±	0.2	0.1	0.0
		Coleoptera	3582	3.1	±	0.0	16.1	11.8	443	2.7	±	0.1	2.6	2.8	619	4.5	±	0.2	7.2	7.4
		Lepidoptera:adult	225	7.2	±	0.3	1.0	1.6	180	10.0	±	0.6	1.0	13.8	63	9.0	±	0.7	0.7	1.1
		larvae	1507	8.6	±	0.2	6.8	8.5	183	6.8	±	0.4	1.1	1.8	86	9.0	±	0.7	1.0	1.0
		Diptera	2681	2.9	±	0.0	12.1	4.1	12917	2.7	±	0.0	74.9	45.3	417	2.8	±	0.1	4.8	1.0
		Miscellaneous/Unknown[2]	871	3.5	±	0.1	3.9	2.4	244	3.6	±	0.2	1.4	2.0	627	3.5	±	0.2	7.2	4.0

[1] May include some Zygentoma from group previously classified as Thysanura.

[2] Approximately half unknown invertebrates and half insects that flew before they could be identified. Additional orders represented by <10. individuals include: Decapoda, Plecoptera, Mantodea, Siphonaptera, Megaloptera, Trichoptera, and Odonata.

Table S3. Model selection results for factors influencing biomass of foliage arthropods by sample (individual plant) and landscape-visit with number of samples as an offset (10–20 samples). To control for spatiotemporal autocorrelation, all models included a crossed random space × time intercept while models at the scale of the sample also included a repeated measure by visit plus observer and plant group. Data for a network of cloud forest sites at 5–6 °S in the Andes of northern Peru (8 landscapes, 2015–2019). Models within ΔAICc <2 were considered equivalent, and we present supported models ranked better than a null model.

	K	AICc	Δ AICc	Weight	Cum.Wt	LL
Individual plant (*n* = 2424 samples)						
New leaves	9	19974.40	0.00	0.62	0.62	-9978.20
Null model	8	19977.60	3.20	0.13	0.75	-9980.80
Time of day	9	19978.30	3.81	0.09	0.84	-9980.10
Elevation	9	19979.00	4.52	0.06	0.90	-9980.40
Wet vegetation	9	19979.50	5.07	0.05	0.95	-9980.70
Fragmentation	9	19979.60	5.11	0.05	1.00	-9980.70
Temperature	8	20028.40	53.91	0.00	1.00	-10006.20
Saturated model	13	20033.00	58.60	0.00	1.00	-10003.40
Landscape phenology (*n* = 104 landscape-visits)						
Null model	4	931.82	0.00	0.33	0.33	-461.60
%Trees w/new leaves	5	932.85	1.03	0.20	0.53	-460.96
%Shrubs w/new leaves	5	933.59	1.77	0.14	0.67	-461.33
%Shrubs + Trees w/new leaves	5	933.98	2.16	0.11	0.78	-461.52
Standardized EVI	5	934.02	2.20	0.11	0.89	-461.54
MODIS EVI	5	934.05	2.23	0.11	1.00	-461.55
Landscape weather (*n* = 116 landscape-visits)						
∩ 90-d Rainfall[1]	6	1513.97	0.00	0.81	0.81	-750.60
∩ 60-d Rainfall[1]	6	1517.57	3.59	0.13	0.94	-752.40
60-d Rainfall	5	1522.04	8.07	0.01	0.96	-755.75
5-d VPDmax	5	1522.16	8.19	0.01	0.97	-755.81
Lat/Long	6	1523.95	9.98	0.01	0.98	-755.59
Leaf-flush phenology[2]	5	1524.54	10.57	0.00	0.98	-757.00
90-d Rainfall	5	1524.73	10.76	0.00	0.98	-757.09
∩ 30-d Rainfall[1]	6	1525.03	11.06	0.00	0.99	-756.13
Null model	4	1525.04	11.06	0.00	0.99	-758.34
Julian date	5	1525.27	11.29	0.00	0.99	-757.36
30-d Rainfall	5	1525.46	11.48	0.00	1.00	-757.46
Elevation	5	1526.61	12.63	0.00	1.00	-758.03
Days since rain	5	1526.95	12.97	0.00	1.00	-758.20
Temperature	5	1527.11	13.14	0.00	1.00	-758.28

[1]∩ represents a curvilinear response to rainfall.
[2]%Trees w/with new leaves, 2016–2019, plus MODIS EVI for 2015.

Table S4. Model selection results for factors influencing leaf flush in cloud forest. Ground based data was scored for a network of cloud forest sites at 5–6 °S in the Andes of northern Peru (8 landscapes, 2015–2019). Satellite EVI data was filtered for forest within a 5-km radius of study sites, 2000–2019. To control for spatiotemporal autocorrelation, models included a crossed random space × time intercept. Models within ΔAICc <2 were considered equivalent, and we present supported models ranked better than a null model.

	K	AICc	Δ AICc	Weight	Cum.Wt	LL
Field observations (*n* = 104 landscape-visits)						
Solar Insolation * Strata	7	1830.74	0.00	1.00	1.00	-908.09
Julian date	5	1844.11	13.37	0.00	1.00	-916.91
Solar Insolation	5	1844.51	13.77	0.00	1.00	-917.11
Julian date * Strata	6	1845.20	14.46	0.00	1.00	-916.39
Day Length	5	1851.44	20.70	0.00	1.00	-920.57
20-day Lux	5	1864.53	33.80	0.00	1.00	-927.12
15-day Rainfall	5	1866.07	35.33	0.00	1.00	-927.88
15-day Lux	5	1866.74	36.00	0.00	1.00	-928.22
20-day Rainfall	5	1867.66	36.93	0.00	1.00	-928.68
Latitude	5	1868.98	38.24	0.00	1.00	-929.34
20-day Temp	5	1869.37	38.63	0.00	1.00	-929.53
20-day VPD	5	1869.96	39.23	0.00	1.00	-929.83
Null Model	4	1870.06	39.33	0.00	1.00	-930.93
15-day VPD	5	1870.89	40.15	0.00	1.00	-930.29
15-day Temp	5	1871.09	40.35	0.00	1.00	-930.39
Elevation	5	1871.52	40.79	0.00	1.00	-930.61
Field observations → MODIS EVI (*n* = 64 landscape-visits)						
%Shrubs + Trees w/new leaves	6	-211.23	0.00	0.34	0.34	112.22
%Shrubs w/new leaves	6	-210.64	0.59	0.25	0.59	111.92
Null model	5	-210.34	0.89	0.22	0.81	110.59
%Trees w/new leaves	6	-210.05	1.18	0.19	1.00	111.63
MODIS EVI (*n* = 2453 landscape-dates)						
Julian + Elevation + Dry season cloud	7	-4404.05	0.00	1.00	1.00	2209.06
Julian date	5	-4392.81	11.24	0.00	1.00	2201.43
Solar insolation	5	-4362.68	41.37	0.00	1.00	2186.36
Elevation + Dry season cloud	6	-4256.32	147.73	0.00	1.00	2134.19
Dry season cloud	5	-4247.38	156.67	0.00	1.00	2128.71
Null model	4	-4244.95	159.10	0.00	1.00	2126.49
Elevation	5	-4244.76	159.29	0.00	1.00	2127.40

[1]∩ represents a curvilinear response to rainfall.

Table S5. Model selection results for response of foliage arthropods to rainfall pre/post the dry season. Landscape visits were grouped before or after lowest 30-d rainfall by landscape each year. Increasing rainfall was considered ≤133 mm in 30 days. Models within ΔAICc <2 were considered equivalent, and we present supported models ranked better than a null model.

	K	AICc	Δ AICc	Weight	Cum.Wt	LL
Wet to drying out (n = 70 landscape-visits)						
∩ 90-d Rainfall[1]	6	899.13	0.00	1.00	1.00	-442.90
∩ 60-d Rainfall[1]	6	911.29	12.17	0.00	1.00	-448.98
Null model	4	915.19	16.06	0.00	1.00	-453.29
90-d Rainfall	5	915.77	16.65	0.00	1.00	-452.42
5-d VPDmax	5	915.89	16.76	0.00	1.00	-452.48
60-d Rainfall	5	916.34	17.22	0.00	1.00	-452.70
Leaf-flush phenology[2]	5	916.93	17.80	0.00	1.00	-452.99
Julian date	5	916.96	17.84	0.00	1.00	-453.01
30-d Rainfall	5	917.50	18.37	0.00	1.00	-453.28
∩ 30-d Rainfall[1]	6	919.24	20.11	0.00	1.00	-452.95
Increasing rain after dry (n = 58 landscape-visits)						
30-d Rain + 5-day VPDmax + Leaf-flush	7	759.14	0.00	0.44	0.44	-371.45
30-d Rain + 5-day VPDmax + Julian date	7	759.14	0.00	0.44	0.88	-371.45
30-d Rainfall	5	763.20	4.06	0.06	0.94	-376.02
∩ 30-d Rainfall[1]	6	764.85	5.71	0.03	0.97	-375.60
60-d Rainfall	5	766.60	7.46	0.01	0.98	-377.72
Julian date	5	766.91	7.78	0.01	0.99	-377.88
Leaf-flush phenology[2]	5	768.34	9.21	0.00	0.99	-378.60
∩ 60-d Rainfall[1]	6	769.09	9.95	0.00	0.99	-377.72
5-d VPDmax	5	769.30	10.17	0.00	1.00	-379.07
90-d Rainfall	5	770.78	11.65	0.00	1.00	-379.82
Null model	4	772.40	13.26	0.00	1.00	-381.82
∩ 90-d Rainfall[1]	6	773.10	13.96	0.00	1.00	-379.72

[1]∩ represents a curvilinear response to rainfall.
[2]%Trees w/with new leaves, 2016–2019, plus MODIS EVI for 2015.

Table S6. Model selection results for factors influencing biomass of aerial insects in cloud forest by sample (individual plant) and landscape-visit with number of samples as an offset (10–20 samples). To control for spatiotemporal autocorrelation, all models included a crossed random space × time intercept while models at the scale of the sample also included a repeated measure by visit plus observer. Data for a network of cloud forest sites at 5–6 °S in the Andes of northern Peru (8 landscapes, 2015–2019). Models within ΔAICc <2 were considered equivalent, and we present supported models ranked better than a null model.

	K	AICc	Δ AICc	Weight	Cum.Wt	LL
Sweep-nets (*n* = 1805 samples)						
Wet vegetation	8	11957.1	0.00	0.88	0.88	-5970.5
Saturated Model	12	11961.4	4.33	0.10	0.98	-5968.6
Null model	7	11966.9	9.81	0.01	0.99	-5976.4
Time of day	8	11968.3	11.16	0.00	0.99	-5976.1
Temperature	8	11968.3	11.22	0.00	0.99	-5976.1
Fragmentation	8	11968.5	11.37	0.00	1.00	-5976.2
Elevation	8	11968.9	11.82	0.00	1.00	-5976.4
Landscape weather (*n* = 101 landscape-visits)						
Since rain + 90-d Rainfall	6	1200.9	0.00	0.73	0.73	-594.0
Since rain + Temperature	6	1204.0	3.09	0.16	0.88	-595.6
Since Rain	5	1204.8	3.84	0.11	0.99	-597.1
5-d VPDmax	5	1212.2	11.24	0.00	0.99	-600.8
90-d Rainfall	5	1212.6	11.66	0.00	0.99	-601.0
Temperature	5	1212.6	11.67	0.00	1.00	-601.0
∩ 90-d Rainfall[1]	6	1213.1	12.16	0.00	1.00	-600.1
Null model	4	1214.6	13.67	0.00	1.00	-603.1
60-d Rain	5	1214.7	13.74	0.00	1.00	-602.0
Julian date	5	1214.9	13.95	0.00	1.00	-602.1
Elevation	5	1216.1	15.16	0.00	1.00	-602.7
30-d Rainfall	5	1216.6	15.69	0.00	1.00	-603.0
∩ 60-d Rainfall[1]	6	1216.9	16.00	0.00	1.00	-602.0
Lat/Long	6	1218.9	17.91	0.00	1.00	-603.0
∩ 30-d Rainfall[1]	6	1218.9	17.95	0.00	1.00	-603.0

[1]∩ represents a curvilinear response to rainfall.

Table S7. Model selection results for factors influencing biomass of leaf-litter invertebrates in cloud forest by sample (individual plant) and landscape-visit with number of samples as an offset (10–20 samples). To control for spatiotemporal autocorrelation, all models included a crossed random space × time intercept while models at the scale of the sample also included a repeated measure by visit plus observer. Data for a network of cloud forest sites at 5–6 °S in the Andes of northern Peru (8 landscapes, 2015–2019). Models within ΔAICc <2 were considered equivalent, and we present supported models ranked better than a null model.

	K	AICc	Δ AICc	Weight	Cum.Wt	LL
	Leaf-litter quadrants (*n* = 1007 samples)					
Temperature	8	8972.2	0.00	0.30	0.30	-4478.0
Time of day	8	8972.6	0.36	0.25	0.56	-4478.2
Elevation	8	8973.5	1.31	0.16	0.72	-4478.7
Null model	7	8973.7	1.51	0.14	0.86	-4479.8
Fragmentation	8	8975.6	3.36	0.06	0.92	-4479.7
Wet vegetation	8	8975.6	3.43	0.05	0.97	-4479.8
Saturated model	12	8977.0	4.76	0.03	1.00	-4476.3
	Landscape weather (*n* = 104 landcape-visits)					
∩ 90-day Rainfall[1]	6	1365.8	0.00	0.36	0.36	-676.5
Null model	4	1367.9	2.07	0.13	0.49	-679.7
Temperature	5	1368.2	2.39	0.11	0.60	-678.8
90-day Rainfall	5	1369.3	3.49	0.06	0.66	-679.4
Elevation	5	1369.4	3.59	0.06	0.72	-679.4
Since Rain	5	1369.7	3.91	0.05	0.78	-679.6
5-d VPDmax	5	1369.9	4.05	0.05	0.82	-679.6
30-d Rainfall	5	1370.0	4.21	0.04	0.87	-679.7
60-d Rainfall	5	1370.0	4.22	0.04	0.91	-679.7
Julian date	5	1370.1	4.27	0.04	0.95	-679.7
Lat/Long	6	1371.0	5.17	0.03	0.98	-679.1
∩ 60-d Rainfall[1]	6	1372.3	6.48	0.01	1.00	-679.7
∩ 30-d Rainfall[1]	6	1374.6	8.77	0.00	1.00	-680.9

[1]∩ represents a curvilinear response to rainfall.

Table S8. Model selection results for factors influencing occurrence of green leaf-chewers in understory foliage branch beating. To control for spatiotemporal autocorrelation, models included a crossed random space × time intercept with a repeated measure by visit plus observer and plant group. Data for a network of cloud forest sites at 5–6 °S in the Andes of northern Peru (8 landscapes, 2015–2019). Models within ΔAICc <2 were considered equivalent, and we present supported models ranked better than a null model.

	K	AICc	Δ AICc	Weight	Cum.Wt	LL
		Lepidoptera larvae (*n* = 1125 individuals)				
Solar insolation	7	1546.33	0.00	0.56	0.56	-766.12
Julian date	7	1548.77	2.44	0.17	0.72	-767.33
90-d Rain	7	1549.78	3.45	0.10	0.82	-767.84
Null model	6	1551.47	5.14	0.04	0.87	-769.70
30-d Rain	7	1551.61	5.28	0.04	0.91	-768.76
Leaf-flush	7	1552.10	5.77	0.03	0.94	-769.00
Sa:Vol Ratio	7	1552.62	6.29	0.02	0.96	-769.26
5-d VPDmax	7	1553.20	6.86	0.02	0.98	-769.55
Cloud cover	7	1553.49	7.16	0.02	0.99	-769.69
Saturated	12	1555.66	9.33	0.01	1.00	-765.69
		Orthoptera (*n* = 512 individuals)				
Null model	6	641.17	0.00	0.18	0.18	-314.50
Sa:Vol Ratio	7	641.38	0.21	0.16	0.35	-313.58
Solar insolation	7	641.41	0.24	0.16	0.51	-313.60
30-d Rain	7	642.15	0.98	0.11	0.62	-313.96
Cloud cover	7	642.43	1.26	0.10	0.72	-314.10
Leaf-flush	7	642.91	1.74	0.08	0.79	-314.35
5-d VPDmax	7	643.13	1.96	0.07	0.86	-314.45
90-d Rain	7	643.15	1.98	0.07	0.93	-314.46
Julian date	7	643.19	2.02	0.07	1.00	-314.48
Saturated	12	650.02	8.85	0.00	1.00	-312.70
		Phasmida (*n* = 129 individuals)				
Leaf-flush	7	185.10	0.00	0.65	0.65	-85.09
Sa:Vol Ratio	7	188.91	3.81	0.10	0.75	-86.99
Null model	6	189.52	4.42	0.07	0.82	-88.42
90-d Rain	7	190.52	5.42	0.04	0.87	-87.80
30-d Rain	7	191.40	6.29	0.03	0.89	-88.24
5-d VPDmax	7	191.63	6.52	0.03	0.92	-88.35
Solar insolation	7	191.68	6.58	0.02	0.94	-88.38
Cloud cover	7	191.75	6.65	0.02	0.97	-88.41
Julian date	7	191.76	6.65	0.02	0.99	-88.42
Saturated	12	193.43	8.33	0.01	1.00	-83.37

SI Desiccation resistance experiments

Arthropod collection. We collected a subset of live foliage arthropods >3 mm as available at the end of each landscape-visit across a range of elevations and months from 2017–2019 (Table S9). We did not include aerial insects (Diptera, Hymenoptera) and we dropped uncommon taxa with insufficient sample size (Dermaptera, Thysanura). Initially, we attempted to conduct experiments for Hemiptera (primarily Cicadellidae) but dropped this group after all individuals died within less than a day. We also dropped a few individuals with evidence of injury (e.g. Orthoptera with missing legs). Arthropods were housed individually without food or water in 2–5 ml plastic vials covered with cheese cloth. Within a few hours of capture, individuals were transported to a centralized location (primarily Chachapoyas ~2400 m, although experiments were occasionally moved to fit field logistics). Here we measured each individual to the nearest 1 mm using calipers (length × width), identified taxa to order, and recorded color. Individual vials were marked with a unique ID number and randomly assigned by order to one of three experimental chambers: low, intermediate, or high humidity (Figure S10).

Experimental set-up. We used readily available materials for experiments. For the three experimental chambers we used large plastic storage containers commonly sold for household use. Humidity was reduced using rechargeable orange (cobalt-chloride free) silica gel beads already in use for drying field equipment. Unlike salts, silica does not maintain a fixed humidity, but slight variability reflected more realistic environmental conditions, and our goal was to understand broad community patterns, not species-specific thresholds. During the experiment individuals were checked every 1–3 days to measure survival time, and we determined death when movement ceased in response to repeated disturbance. Occasional longer times between checks were required because of field logistics (e.g. camping trips to remote sites), and all chambers were checked on the same schedule. For checks >1 day apart we used a midpoint between checks as the date of death.

Controlling for arthropod size. We calculated approximate surface-area-to-volume ratios for individual arthropods based on length (*l*) and width (*w*) assuming a cylindrical shape:

$$SA\!:\!Vol = \frac{\pi w(l\ +\ 0.5w)}{\pi w l}$$

Mean SA: Vol ratio (1.7–2.4x) and width (2.0–3.2 mm) were relatively similar with a range of sizes represented for each taxon, although taxa differed in length (Table S10). At a mean SA: Vol ratio of 2.0x and VPD of zero, predatory Araneae survived 2x longer at 15 days than other insect orders which were relatively similar (5.9–7.7 days). We present arthropod orders with the largest sample sizes in the main text (n = 35–140). We found the same directional effect for Blattodea, but with reduced power (n = 26) effects of VPD were only marginally significant (P = 0.10).

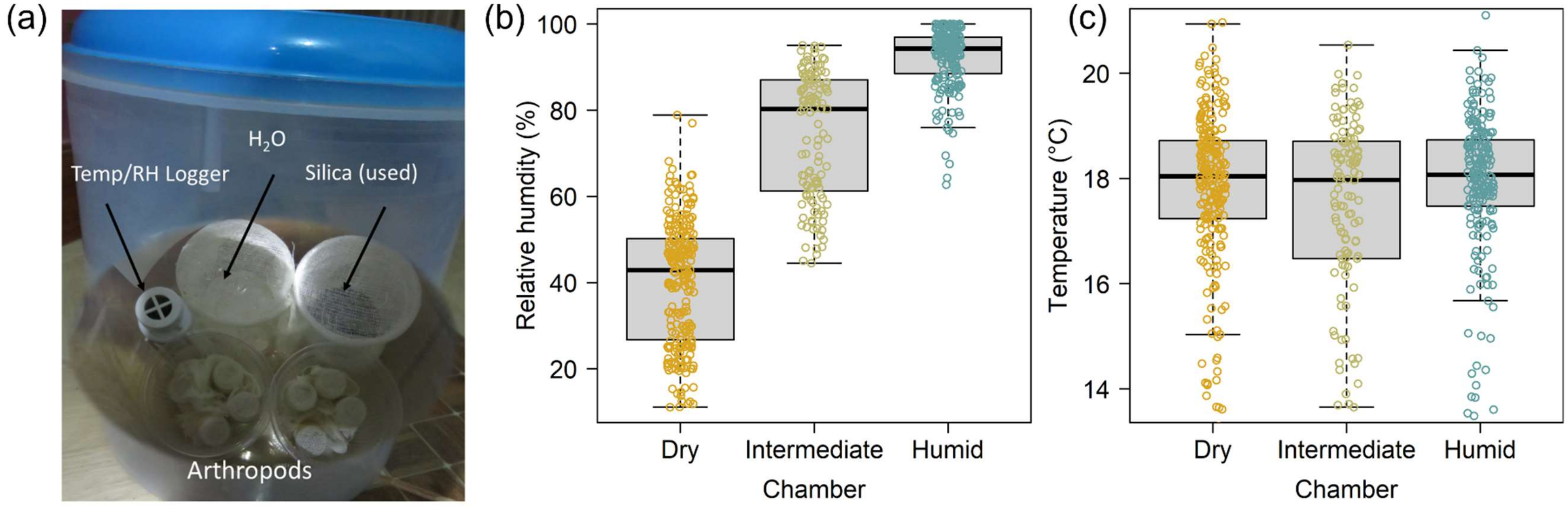

Figure S10. Simple experiment used to measure desiccation resistance across cloud forest arthropod taxa. (a) Humidity was reduced in experimental chambers using a range of new to used silica and monitored using Hobowear data loggers (U23-001); the humid control also included used silica and water. (b) Mean daily relative humidity and (c) temperature in sealed chambers during experiments, 2017--2019.

Table S9. Summary of arthropod taxa collected across different elevations and months for desiccation resistance experiments; individuals were randomly assigned to one of three experimental chambers with different relative humidity (RH). Arthropods >3 mm were collected as available from branch beating of understory foliage across a network of 7 cloud forest landscapes in northern Perú, 2017–2019.

	Dry = 40% RH							Intermediate = 74% RH							Wet = 92% RH						
	1850 m	2010 m	2310 m	2510 m	2740 m	2780 m	3100 m	1850 m	2010 m	2310 m	2510 m	2740 m	2780 m	3100 m	1850 m	2010 m	2310 m	2510 m	2740 m	2780 m	3100 m
Araneae (*n* = 59 individuals)																					
Jan		1			2		1		2			2		4		1			2		1
Feb						1							1								
May				1		1	1						1	1		1			1		1
Jun	1		4			1		4		1							3				
Jul			1	1			1			2		3		1			3		1		1
Sep		1				1															
Nov					1											1				1	
Orthoptera (*n* = 86 individuals)																					
Jan					1				4			4							1		
Feb						1							2								
May		1				1		1	2		1		1			1		1		1	
Jun	1		1	1				4		2					1		2	1			
Jul			9		3		1			7	4	3					5		1		
Sep		1														1	2			2	1
Oct																	1			1	
Nov	2				2	2										1				1	
Blattodea (*n* = 25 individuals)																					
Jan					1									1					1		
Feb						1							2							1	
May		1											1								
Jun			1			1				3							1			1	
Jul										1		1					2		1		
Sep		1															1				
Oct							1														
Nov					1																
Phasmida (*n* = 35 individuals)																					
Jan					1		1					2		1					1		1
May				1	1	1					1		1					1	1	1	
Jun	1			1		1		1					1		1			2			
Jul			1				1			1	2	2							1		
Sep																				2	
Oct			1																	1	
Coleoptera (*n* = 64 individuals)																					
Jan		2			2		1		5			2		1		2			2		
May		1																	1		
Jun	1			1		1		5		1								1		2	
Jul			1	1						2	1	4					3	1			
Sep							1										3				1
Oct				2		2														2	
Nov	4				2	1										1				1	

	Dry = 40% RH							Intermediate = 74% RH							Wet = 92% RH						
	1850 m	2010 m	2310 m	2510 m	2740 m	2780 m	3100 m	1850 m	2010 m	2310 m	2510 m	2740 m	2780 m	3100 m	1850 m	2010 m	2310 m	2510 m	2740 m	2780 m	3100 m
Lepidoptera larvae, green coloration (*n* = 68 individuals)																					
Jan		1							1					1							
Feb						1															
May	1	1		2		1	3				3	2		1				2	6	1	
Jun			1			2		1					1					1		1	
Jul				2	3		1			1	2			5				2	1		3
Sep																	1			1	
Oct							2										1	2		1	1
Nov	1				1	1														2	
Lepidoptera larvae, melanistic coloration (*n* = 140 individuals)																					
Jan							2		7			1		3		1					2
Feb													2								
May		2		1		1	2	1			1		3	3	1			2	1	2	4
Jun	1		1	2		2		2		3			1				3	2		1	
Jul			7	1	5		7			5	5	7		10			2	2			6
Sep																2	3				
Oct						2											5	2		1	1
Nov					2		1									4					

Table S10. Parameter estimates from experimental test of desiccation resistance for six arthropod taxa in cloud forest. Model intercepts represent survival days at a mean surface-area-to-volume ratio of 2.0 and vapor pressure deficit of zero. Estimates are on the response scale.

		Length (mm)			Model Results						
Taxa	*n*	Mean	±	SE	Parameter	Estimate	LCI	UCI	*t*	*P*	R^2
Araneae	59	6.2	±	0.3	Intercept	14.98	10.79	21.16	17.9	<0.001	-
					VPD (kPa)	-0.46	-0.64	-0.20	-3.2	0.002	0.14
					SA: Vol Ratio	-0.30	-0.49	-0.04	-2.3	0.023	0.07
Orthoptera	86	8.5	±	0.5	Intercept	5.91	4.83	7.25	22.0	<0.001	-
					VPD (kPa)	-0.42	-0.53	-0.28	-5.0	<0.001	0.24
					SA: Vol Ratio	-0.26	-0.38	-0.12	-3.4	0.001	0.10
Blattodea	25	7.0	±	0.5	Intercept	7.30	4.13	13.22	8.4	<0.001	-
					VPD (kPa)	-0.37	-0.65	0.16	-1.7	0.101	0.11
					SA: Vol Ratio	0.14	-0.40	1.26	0.4	0.686	0.02
Phasmida	35	22.7	±	1.4	Intercept	7.72	6.10	9.82	20.4	<0.001	-
					VPD (kPa)	-0.39	-0.53	-0.19	-3.6	0.001	0.29
					SA: Vol Ratio	-0.18	-0.37	0.05	-1.6	0.118	0.14
Coleoptera	64	8.1	±	0.5	Intercept	6.93	5.01	9.68	13.7	<0.001	-
					VPD (kPa)	-0.52	-0.64	-0.35	-4.7	<0.001	0.30
					SA: Vol Ratio	-0.13	-0.31	0.11	-1.0	0.302	0.05
Lepidoptera	68	13.6	±	0.8	Intercept	6.35	5.26	7.68	24.9	<0.001	-
Green larvae					VPD (kPa)	-0.38	-0.50	-0.24	-4.7	<0.001	0.23
					SA: Vol Ratio	-0.18	-0.29	-0.05	-2.6	0.011	0.07
Lepidoptera	140	12.5	±	0.5	Intercept	6.31	5.42	7.36	29.9	<0.001	-
Tan/brown/black larvae					VPD (kPa)	0.03	-0.13	0.23	0.4	0.708	0.00
					SA: Vol Ratio	-0.18	-0.27	-0.08	-3.3	0.001	0.07

SI Arthropod biomass model

Modeling approach. We used field parameter estimates to describe dynamic response of foliage arthropods to rainfall fluctuations around intermediate optima. Our modeling approach included the following steps: (1) We modeled 30-day rainfall seasonality by landscape and year using generalized additive mixed models (GAMMs) with a cyclical cubic regression spline. We extracted Julian dates for point of trough (POT) and peak (POP); we used POT dates by landscape-year to reflect spatiotemporal variability during the dry season, and for POP we used a mean date for the region (2) Before POT dates we used biomass estimates from a curvilinear relationship with 90-day rainfall accumulation. On a 30-day time-step biomass peaked at 133 mm which we defined as optimal rainfall. (3) After POT dates we used biomass estimates from a linear relationship with 30-day rainfall (before regional POP and less than optimal rainfall). (4) Rainfall-derived estimates of arthropod biomass were then used as input data to examine variability and model phenological means on multidecadal time scales across a rainfall gradient.

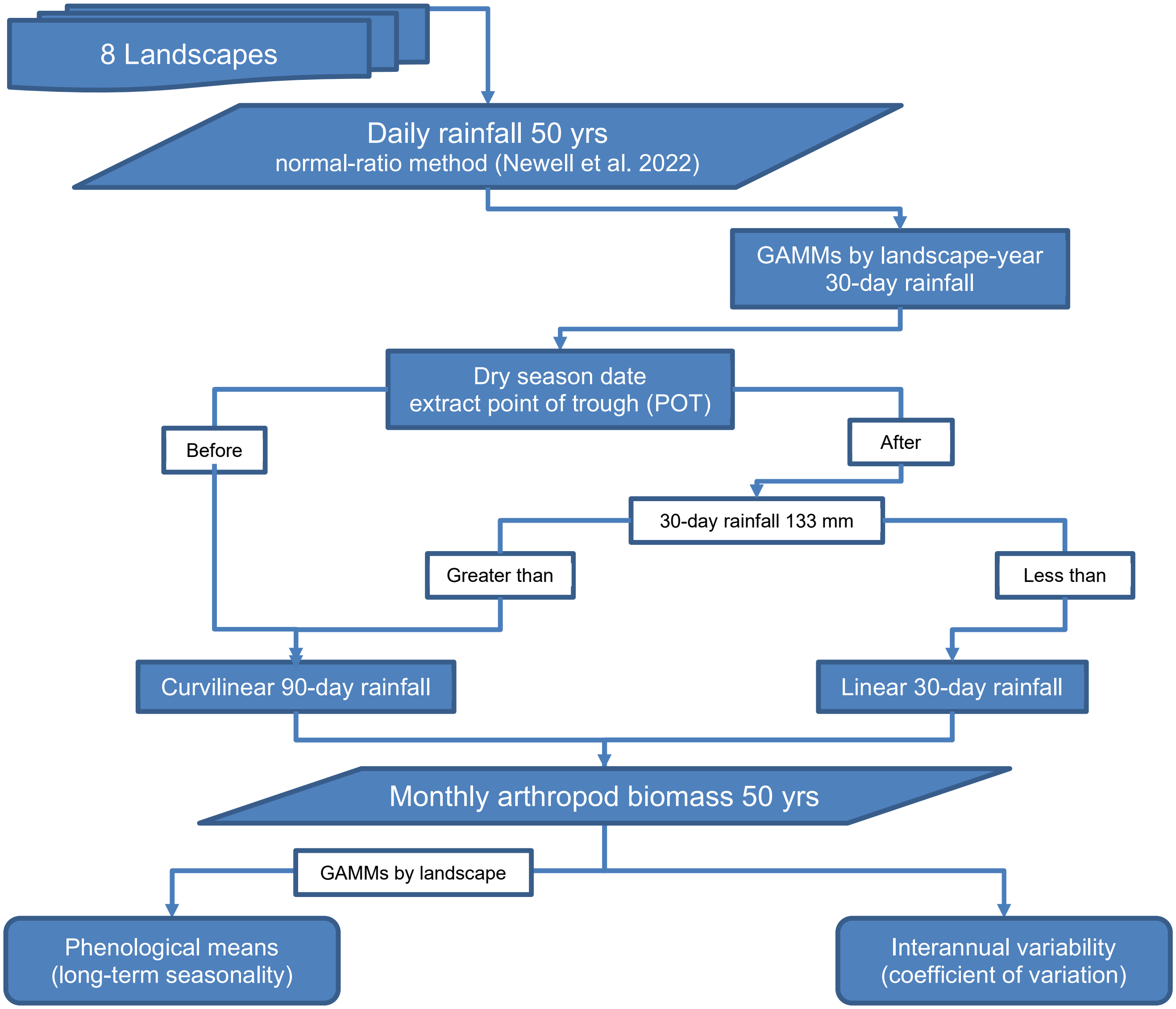

Figure S11. Flowchart of data inputs, analytical processes, and data outputs for the intermediate rainfall model used to predict arthropod biomass across a network of 8 cloud forest landscapes in northern Peru.

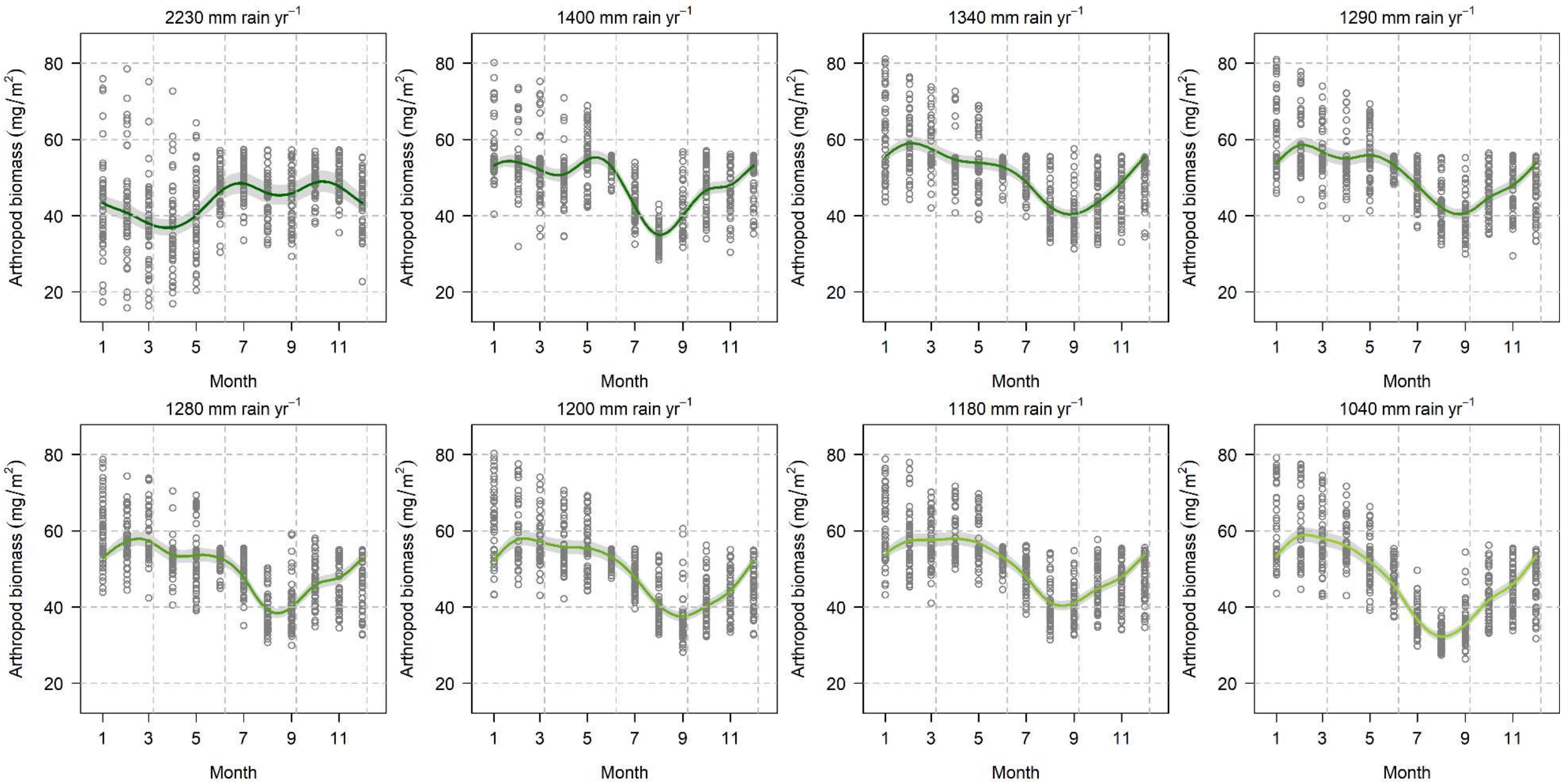

Figure S12. Multidecadal phenological means for arthropod biomass by landscape as predicted by the intermediate-rainfall model using 50 years of regional data scaled to *in situ* rain gauges. Cloud forest network at 5–6 °S in the Andes of northern Peru ordered by mean annual rainfall estimated for the same period.

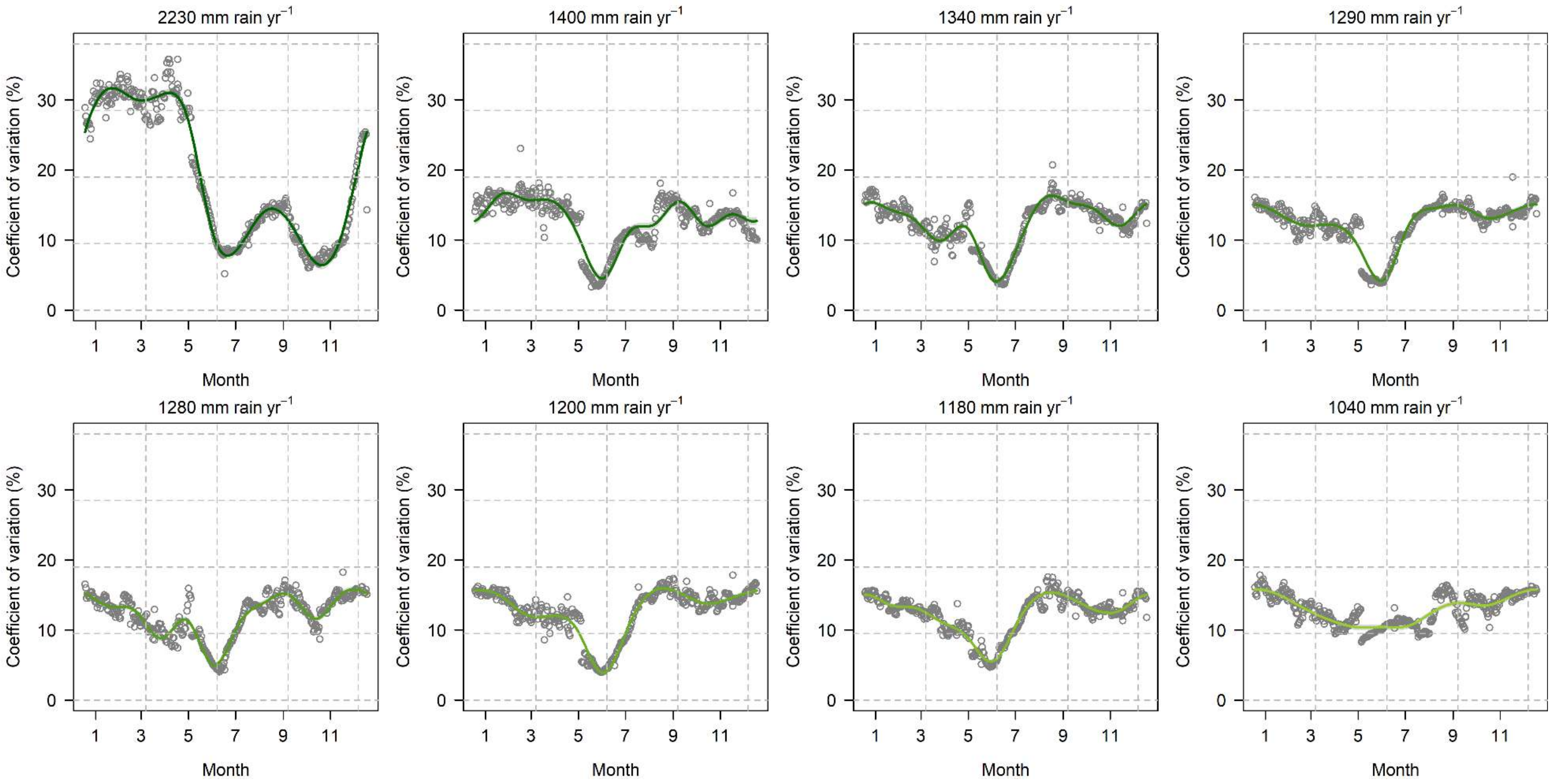

Figure S13. Coefficient of variation for arthropod biomass by landscape as predicted by the intermediate-rainfall model using 50 years of regional data scaled to *in situ* rain gauges. Cloud forest network at 5–6 °S in the Andes of northern Peru ordered by mean annual rainfall estimated for the same period.

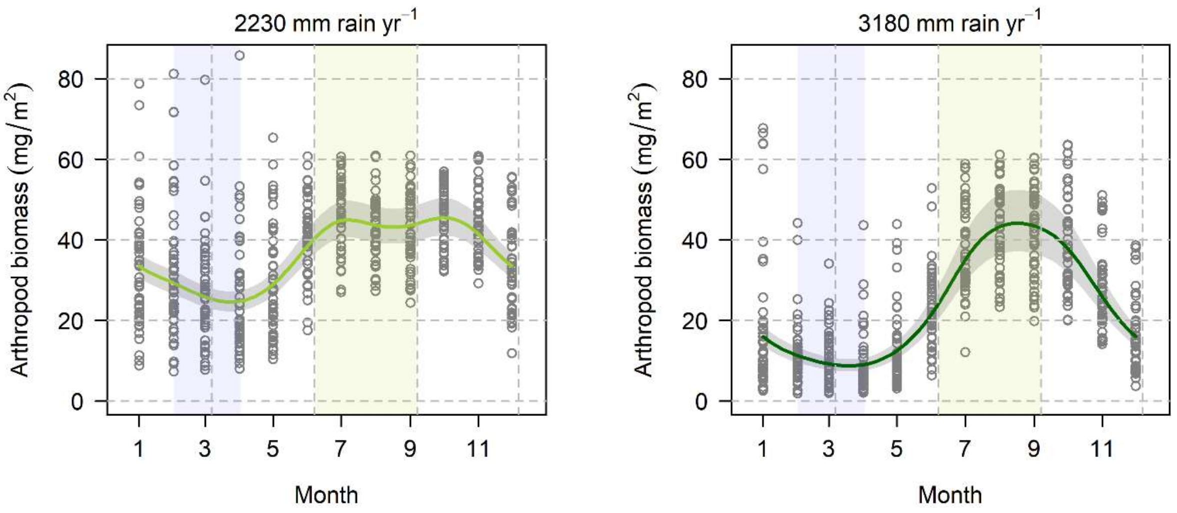

Figure S14. Arthropod biomass model incorporating rainfall uncertainty at the wettest site on the eastern slopes of the Andes (BPAM, Venceremos guard station). As rainfall increased, the multi-decadal model predicted dry season resource maxima were accentuated by reduced wet season biomass.

SI Project collaborators in Perú

LANDHOLDERS

La Red de Áreas de Conservación Privada de Amazonas (Red AMA).

José de la Torre, presidente**,** Perico Heredia, vicepresidente**,** Leyda Gueiler Rimarachín Cayatopa, secretaria.

Área de Conservación Privada de Bosque Berlín, Bagua Grande.

Leyda Gueiler Rimarachín Cayatopa Ricardo Rimarachín, Carmela Cayatopa, Antonio Rojas

Comunidad Campesina de Beirut, Corosha, Bongára.

Edwin Guiva, presidente, Blanca Guivin, Carmen Guiva, Demasio Edkin y Teodora, Homero "Pancho" Lopez, Lucas Vegas, Marcos Guadelupe, Maria Llaja

Área de Conservación Privada de Huiquilla, Choctámal.

José de la Torre, Miguel Cruz

Comunidad Campesina de Levanto.

Presidentes de la comunidad: Asuncion Torres, Emerehildo Salombavilde, Don Santos, y Alejandro Cruz

Comunidad Campesina de Mayno.

Don Teodoro, presidente de la comunidad

Fundo Ecológico Don Diego, Pomacochas, y Nuevo Gualulo.

Dario Cotrina, Diego Poclín Tuesta, Ernesto Arajo, Francisca Tuestadet, Joel Poclín, Doña Juana, Paulino Arajo

Área de Conservación Privada Bosque de Palmeras de Ocol, Molinopampa.

Elmira Muñoz Jil, Irma Cruz Viaya, Marleny Servan Cruz, Nixon Pilco Melendez, Orfita Mori Cachay, Pablo Mori Cachay, Patricia Rimachi Diaz, Rosa Amelia Diaz Perea

Comunidad Campesina de San Lorenzo.

Aparicio Medina Vasquez, presidente de la comunidad, Efijenio Katpo, Juan José Chicano, Kelmar Valle, Secundino Rojas, Vincente Chavez y esposa.

Comunidad Campesina de Velapata.

Mario Tuesta Vargas y familia

FOOD, LODGING, & GUIDES

Comunidad Campesina de Beirut, Corosha, Bongára.

Asociación Turística de Beirut: Adelayda Chosguild Tioloc, Alberto Saul Benavides Gonzales, Bernardina Mirano Mas, Graciola Campos de la Cruz, Jorge Juarez, Judith Oyarce Ehasquibol, Lizeth Arista Aybar, Lucas Vega Jr., Maria Herlit Goñas Sopla, Miriarb Portacarrero Lápiz, Neli Montenegro Llaja, Rosario Gómez Montenegro, Sarai Cieza Goñaas, Silvia Valle Rojas, Victor Ramos, Yuly Rojas Diaz, Zoilita del Pilar Torres Jauregui, Zoyla Marina Goñas de Cieza

Área de Conservación Privada de Bosque Berlín, Bagua Grande.

Leyda Gueiler Rimarachín Cayatopa, Victoria Rimarachín Cayatopa

Área de Conservación Privada de Huiquilla, Choctámal.

Alejandro Cuipal Chuquisuta, Alsira Chavez Iop, Alejo and Jonny Cuipal Chavez

Comunidad Campesina de Levanto.

Jorge Mendoza

Fundo Ecológico Don Diego, Pomacochas and Nuevo Gualulo.

Diego Poclín Pinedo, Elda Diaz Gamuro

Área de Conservación Privada Bosque de Palmeras de Ocol, Molinopampa.

Asociación de Las Mujeres de ACP Palmeras de Ocol: Dani Juanita Diaz Rojas, Diana Carina Mori Servan, Ena Carmela Tamallo Diaz, Graciela Tafur Maldonado, Marlene Servan Cruz, Marta Rojas Gomez, Olger Callilgos Acosta, Pablo Mori Cachay, Pablito Mori Servan, Patricia Rimachi Diaz, Rosa Amelia Diaz Perea, Teunilo Calampa Santijan, Wilmer Vicaya Sopla

Comunidad Campesina de San Lorenzo.

Felicita Vasquez Altamirano, Idelfia Ruiz Villanueva, Rodolfo Banda, Wilson Loaisa y esposa

Comunidad Campesina de Velapata.

Genarita Cruz y pareja

Bosque de Protección Alto Mayo (BPAM), Venceremos guard station.

Silvia Ivonne Paico Vera, Jhonny Ramos, Jolo Elias Cercado Cabrera, Jorge Luis Altantara Veía, and guardeparques.

TRANSPORTATION

Anival (Levanto), Cleo (Choctamal), Dago (Bagua Grande), Humberto Vilca, Jhonny Cuipal (Choctámal), Justino (Choctámal), Orlando (Levanto), Orlando Cruz (Choctámal), Noe (Chacha).

PERMITS

Diego García Olaechea (CORBIDI), Silvia Ivonne Paico Vera (BPAM), Lizeth Natali Cayo Rodriguez (SERFOR), Marco A. Enciso (SERFOR), Priscilla Pellisier (CORBIDI), Thomas Valqui (CORBIDI).

WEATHER DATA

Ivonne Paico Vera & park guards (BPAM), Wagner Guzman Castillo (IIAP), Períco Heredia Arce (Red AMA), Rolando Salas López & Elgar Barboza Castillo (Universidad Toribio Rodriguez de Mendoza INDES-CES). Communities & Red AMA: Homero Francisco Lopez, Maria Llaja; Diego Poclín Pinedo, Diego Poclín Tuesta, Elda Díaz Guamuro, Vincente Chavez & family, Leyda Gueiler & Victoria Rimarachín Cayatopa, Ricardo Rimarachín, Camila Cayatopa, Pablo Mori Cachay, Marlene Servan Cruz, Diana Carina & Pablito Mori Servan, Humberto & Segundo Vilca, Jose La Torre, Alsirra Cuipal Chuquisuta, Alejo Chavez.

OTHER

Antonio García Bravo (CORBIDI), Emperatriz Muñoz, Fernando Angulo Pratolongo (CORBIDI), Imanol Martin Gonzalez, Karen Marie Pedersen (Peace Corps), Lizette Mendez Fasabi, Maddy Stokes (Peace Corps), Marina Cruz Santíllán Trigoso, Perico Heredia Arce, Wagner Guzmán Castillo, José Llaja Soplin & Dona Regalado Fernandez (Chachapoyas Backpackers), Carlos Altamirano.
